# Bacterial and Host Determinants of MAL Activation upon EPEC Infection: The Roles of Tir, ABRA, and FLRT3

**DOI:** 10.1371/journal.ppat.1001332

**Published:** 2011-04-07

**Authors:** Robert J. W. Heath, John M. Leong, Balázs Visegrády, Laura M. Machesky, Ramnik J. Xavier

**Affiliations:** 1 Center for Computational and Integrative Biology, Massachusetts General Hospital, Harvard Medical School, Boston, Massachusetts, United States of America; 2 Gastrointestinal Unit, Center for Inflammatory Bowel Disease, Massachusetts General Hospital, Harvard Medical School, Boston, Massachusetts, United States of America; 3 Department of Molecular Genetics and Microbiology, UMass Medical School, Worcester, Massachusetts, United States of America; 4 Beatson Institute for Cancer Research, Garscube Estate, Bearsden, Glasgow, United Kingdom; 5 The Broad Institute of MIT and Harvard, Cambridge, Massachusetts, United States of America; University of Helsinki, Finland

## Abstract

Infection of host cells by pathogenic microbes triggers signal transduction pathways leading to a multitude of host cell responses including actin cytoskeletal re-arrangements and transcriptional programs. The diarrheagenic pathogens *Enteropathogenic E. coli* (EPEC) and the related *Enterohemorrhagic E. coli* (EHEC) subvert the host-cell actin cytoskeleton to form attaching and effacing lesions on the surface of intestinal epithelial cells by injecting effector proteins via a type III secretion system. Here we use a MAL translocation assay to establish the effect of bacterial pathogens on host cell signaling to transcription factor activation. MAL is a cofactor of Serum response factor (SRF), a transcription factor with important roles in the regulation of the actin cytoskeleton. We show that EPEC induces nuclear accumulation of MAL-GFP. The translocated intimin receptor is essential for this process and phosphorylation of Tyrosine residues 454 and 474 is important. Using an expression screen we identify FLRT3, C22orf28 and TESK1 as novel activators of SRF. Importantly we demonstrate that ABRA (actin-binding Rho-activating protein, also known as STARS) is necessary for EPEC-induced nuclear accumulation of MAL and the novel SRF activator FLRT3, is a component of this pathway. We further demonstrate that ABRA is important for structural maintenance of EPEC pedestals. Our results uncover novel components in pathogen-activated cytoskeleton signalling to MAL activation.

## Author Summary

Many significant immune diseases are caused by bacterial pathogens that deliver effector proteins into their host. The pathogen uses these proteins to subvert the hosts' normal cytosolic defense in a way that services the pathogen. It is therefore important to understand the normal processes of a cell and how they are affected by bacterial infection. We have established the effect of bacteria on host cell signalling to the transcription factor serum response factor. Serum response factor is a widely expressed transcription factor that controls the expression of many important genes. We show that Enteropathogenic *E. coli* infection can activate serum response factor and that the effector protein Tir is essential for this activation. Furthermore, we identify new genes that are important in this infection-induced activation and show that they are important in maintaining structures necessary for Enteropathogenic *E. coli* infection.

## Introduction

Infection of host cells by pathogenic microbes triggers signal transduction pathways leading to a multitude of host cell responses including actin cytoskeletal re-arrangements and transcriptional programs. This is achieved via the delivery of virulence factors directly into target cells [1]. Often structurally divergent, these effector proteins mimic eukaryotic functions [2] and are usually delivered into the host-cell cytosol by needle-like, type III (T3SS), type IV (T4SS) and type VI (T6SS) secretion systems [3]. These secretion systems are large multi-protein complexes that span the entire cell envelope. More than 25 species of Gram-negative bacteria have a Type III secretion system [4]. Many of the T3SS secreted bacterial virulence factors seem to fall into two general classes: 1) those that indirectly subvert actin dynamics by modulating the host-cell machinery involved in actin organization, or 2) those that directly bind actin [3]. Although the types of virulence factors introduced by various organisms differ, there is a shared theme of the subversion of nucleation promoting factors directly or indirectly via Rho, Rac or Cdc42.

Bacterial pathogens can manipulate a host-cell's cytoskeleton to attach, invade and/or move in the cell. A conserved strategy involves manipulating F-actin by modulating or mimicking G proteins in the host cell. Among transcription factors, Globular (G)-actin to Filamentous (F)-actin changes are sensed by serum response factor (SRF). SRF is a widely expressed transcription factor that controls the expression of many immediate early, muscle-specific and cytoskeletal genes [5], [6]. The activity of SRF is primarily controlled by its interaction with signal-regulated or tissue-specific regulatory cofactors. Two families of signal-regulated cofactors have been identified: the ternary complex factor (TCF) family, which are activated by mitogen activated protein (MAP) kinase phosphorylation [7], and the myocardin-related transcription factors (MRTFs). The MRTFs include Myocardin, MAL (also known as MRTF-A, BSAC or MKL1) and MRTF-B (also called MKL2 or MAL16). Rho-family GTPases and monomeric actin regulate the activity of MAL and MRTF-B [8], [9]. Rho family-mediated changes in actin dynamics are sensed by MAL, which contains G-actin-binding RPEL motifs at the N-terminus. Stimulation of Rho family-GTPases releases MAL from an inhibitory complex with G-actin and strongly activates SRF-regulated transcription [9], [10].

When overexpressed in heterologous systems, a number of wild type proteins involved in RhoGTPase signalling to actin dynamics, including Cdc42, Rac and VASP can activate SRF [8], [11]. However, these results have not been explored in the context of a potential link between bacterial pathogenesis and SRF mediated transcriptional programs. Furthermore understanding actin biology in the context of pathogen triggers also offers insight into regulation of the actin machinery in the host cell. This has previously proven to be a very successful avenue, especially for the study of bacterial factors targeting host cell GTPases. Both cellular and microbiological approaches have brought great insight into the bacterial infection process and host physiology [12], [13].

We developed a screen to identify both bacterial and host-cell factors important for pathogenesis. We use MAL-GFP translocation to establish the effect of bacterial pathogens on actin-mediated, host cell signalling to transcription factors and identify novel host cell factors involved in the maintenance of the EPEC pedestal. Here we report that EPEC infection induces nuclear accumulation of MAL-GFP and subsequent transcription of SRF target genes, in a manner dependent on pedestal formation. The translocated EPEC effector Tir is essential, as is phosphorylation of Tir by host cell kinases. We show that the host gene ABRA (also known as STARS), is necessary for MAL translocation and that FLRT3 is a novel SRF activator that functions as a signalling intermediary between the pedestal and nucleus.

## Results

### EPEC but not AIEC, *S.* Typhimurium, or *E. coli* K12, causes nuclear accumulation of MAL-GFP

**Figure 1:**
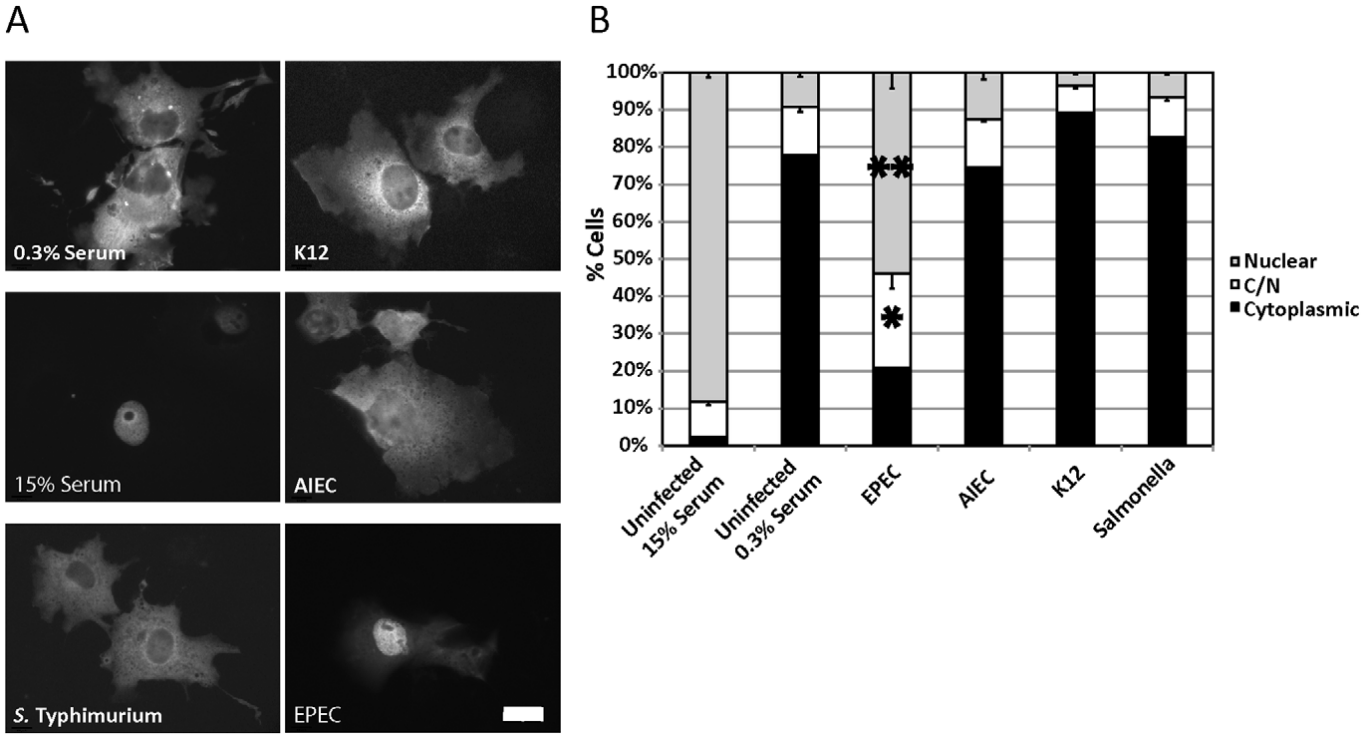
EPEC induces MAL-GFP nuclear accumulation. A. EPEC induces nuclear accumulation of MAL-GFP. COS-7 cells were transfected with MAL-GFP, serum starved then stimulated with 15% foetal calf serum (FCS) or infected with the indicated bacteria. B. The percentage of transfected cells from panel A that had MAL-GFP in the nucleus, cytoplasm or both nucleus and cytoplasm was determined. Data are the means of at least 3 experiments where a minimum of 150 transfected cells were counted for each condition of each experiment. Values are means ± SEM. **P = 7.31336^−05^, *P = 0.027. Scale Bar = 20 µm.

SRF activation through the co-factor MAL requires Rho-mediated actin signalling [8]. G-actin binds directly to MAL [9]; extracellular stimuli activate cellular GTPases (Rho, Rac and CDC42) driving actin polymerization and altering the G-/F-actin ratio. This releases MAL, allowing it to accumulate in the nucleus, form a complex with SRF and drive transcription. In many cell types MAL is predominantly cytoplasmic and accumulates in the nucleus only upon stimulation to activate target genes [9], [14]–[16]. Using MAL nuclear accumulation as a readout we developed a microscopy-based screen for SRF activation in epithelial cells (Figure 1A).

To test the effects of bacterial infection on the regulation of the actin cytoskeleton, we screened a panel of gastrointestinal tract-associated bacterial pathogens including Enteropathogenic *E. coli* (EPEC), Adherent Invasive *E. coli* (AIEC), *Salmonella enterica* serovar Typhimurium (*S.* Typhimurium), and a non-pathogenic *E. coli* K12 for the ability to induce the nuclear accumulation of the SRF co-factor MAL. COS-7 cells were transfected with MAL-GFP. After 18 hours, the transfected cells were serum starved for a further 24 hours and then infected in DMEM containing 0.3% Foetal Calf Serum (FCS) with bacteria for a total of 5 hours. Following infection, cells were washed, fixed and stained for immunofluorescence. We found that EPEC, but not AIEC, K12 or *S.* Typhimurium could induce robust nuclear accumulation of MAL-GFP (Figure 1A). The percentage of cells exhibiting nuclear localization and both cytoplasmic and nuclear (C/N) localization of MAL-GFP increased significantly from 9.19%±1.09% to 53.94%±4.21%, and from 13%±1.36% to 25.23±4.02% respectively, when compared to the uninfected 0.3% serum control (Figure 1B). The nuclear localization induced by EPEC was less efficient than that of the 15% serum control (Figure 1A and B). These data suggest that nuclear localisation of MAL is specific to EPEC infection and not merely a general response to host/pathogen interaction or actin-mediated invasion events.

### SRF is necessary for EPEC induced MAL-GFP accumulation in the nucleus

**Figure 2:**
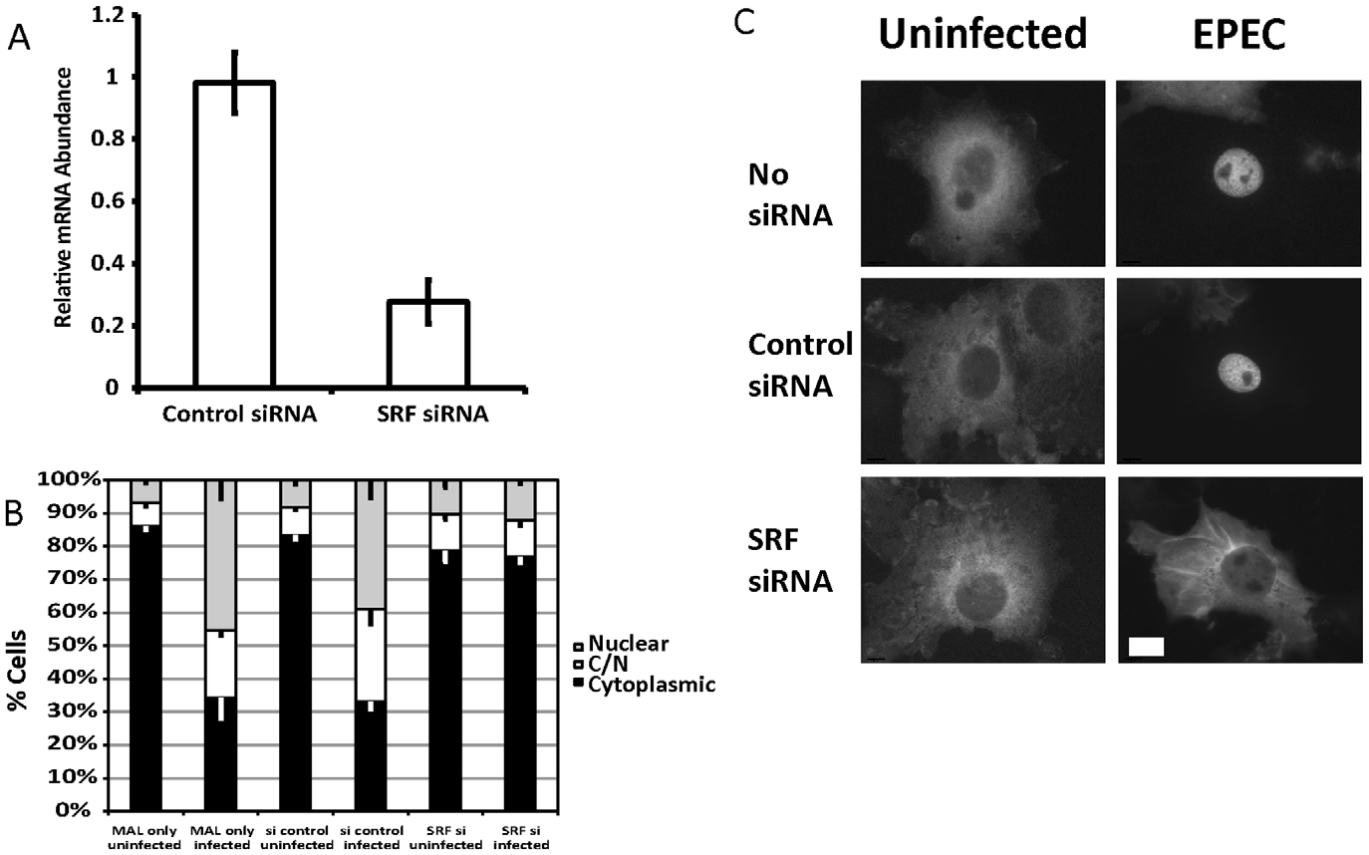
SRF is important for EPEC induced MAL-GFP translocation. A. COS-7 cells transfected with siRNA targeting SRF or a non-targeting siRNA. After 72 hours knockdown efficiency was assessed using real-time quantitative RT-PCR with SRF specific primers and normalized to *GAPDH*. Shown are means of three experiments ± standard deviation. B. Infection of SRF-knockdown cells with EPEC results in a significant reduction in nuclear accumulation of MAL-GFP, P = 0.0081 relative to the no siRNA control. Data are the means of at least 3 experiments where a minimum of 150 transfected cells were counted for each condition of each experiment. Values are means ± SEM. C. Representative images of cells counted in panel B. Scale Bar = 20 µm.

MAL (MRTF-A) is a well-described cofactor for Serum Response Factor (SRF). We wanted to confirm that the EPEC-induced nuclear localization of MAL-GFP was actually associated with SRF. To test this we transfected COS-7 cells with siRNA targeting SRF or a non-targeting control siRNA (Invitrogen), and determined the knockdown efficiency by quantitative RT-PCR (Figure 2A).

Infection of SRF-knockdown cells with EPEC resulted in a significant reduction in nuclear accumulation of MAL-GFP to 13.39%±1.61% compared to infection of wild type or non-targeting siRNA transfected COS-7 cells 45.38%±5.5% and 39.04%±5.39% respectively (Figure 2B and C). It is likely that SRF knockdown affects cytoskeletal gene expression, which in turn affects MAL localization.

### SRF target genes are activated by EPEC infection

**Figure 3:**
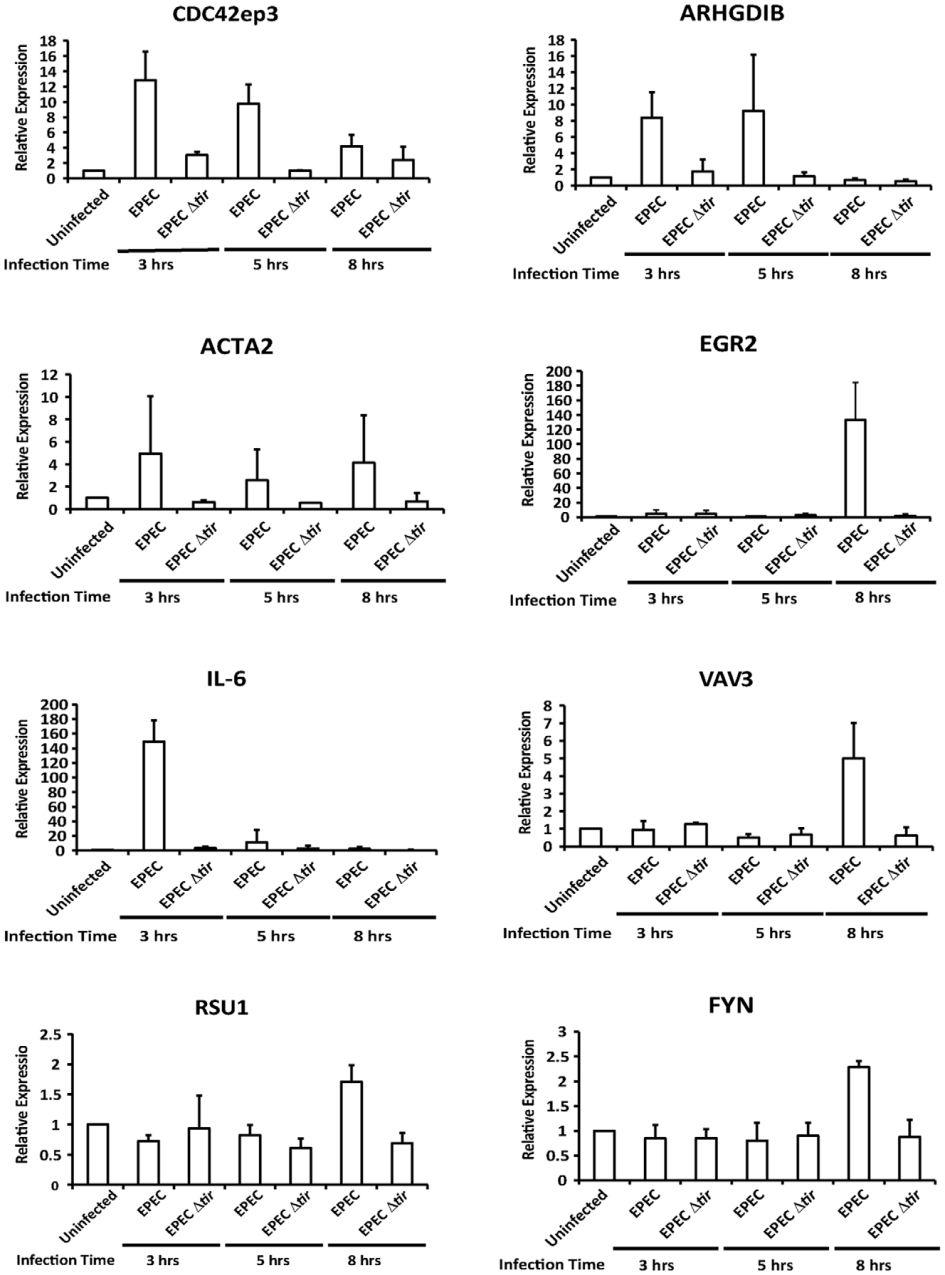
Transcription of SRF target genes is activated by EPEC infection. Transcription of SRF target genes measured by quantitative polymerase chain reaction (qRT-PCR). Data are the means of at least 3 experiments ± standard deviation.

To confirm that MAL functions as a coactivator of SRF during EPEC infection we measured the expression levels of known SRF target genes at 3, 5 and 8 hours post infection (Figure S1). Of those tested *Cdc42ep3* (CDC42 effector protein), *ARHGDIB* (Rho GDP dissociation inhibitor (GDI) beta), *Acta2* (Alpha actin 2), *Egr2* (Early growth response 2), *IL-6* (Interleukin 6) and *Vav3* (vav 1 guanine nucleotide exchange factor), were induced by EPEC infection but not by infection with EPEC Δ*tir* (Figures 3 and S1). *Fyn*, *Rsu1 and c-fos* were not activated by EPEC infection during the timepoints measured. This data supports the hypothesis that nuclear MAL functions as an SRF cofactor during EPEC infection.

### EPEC-induced MAL-GFP translocation requires Tir and is dependent on phosphorylation of Y454 and Y474

**Figure 4:**
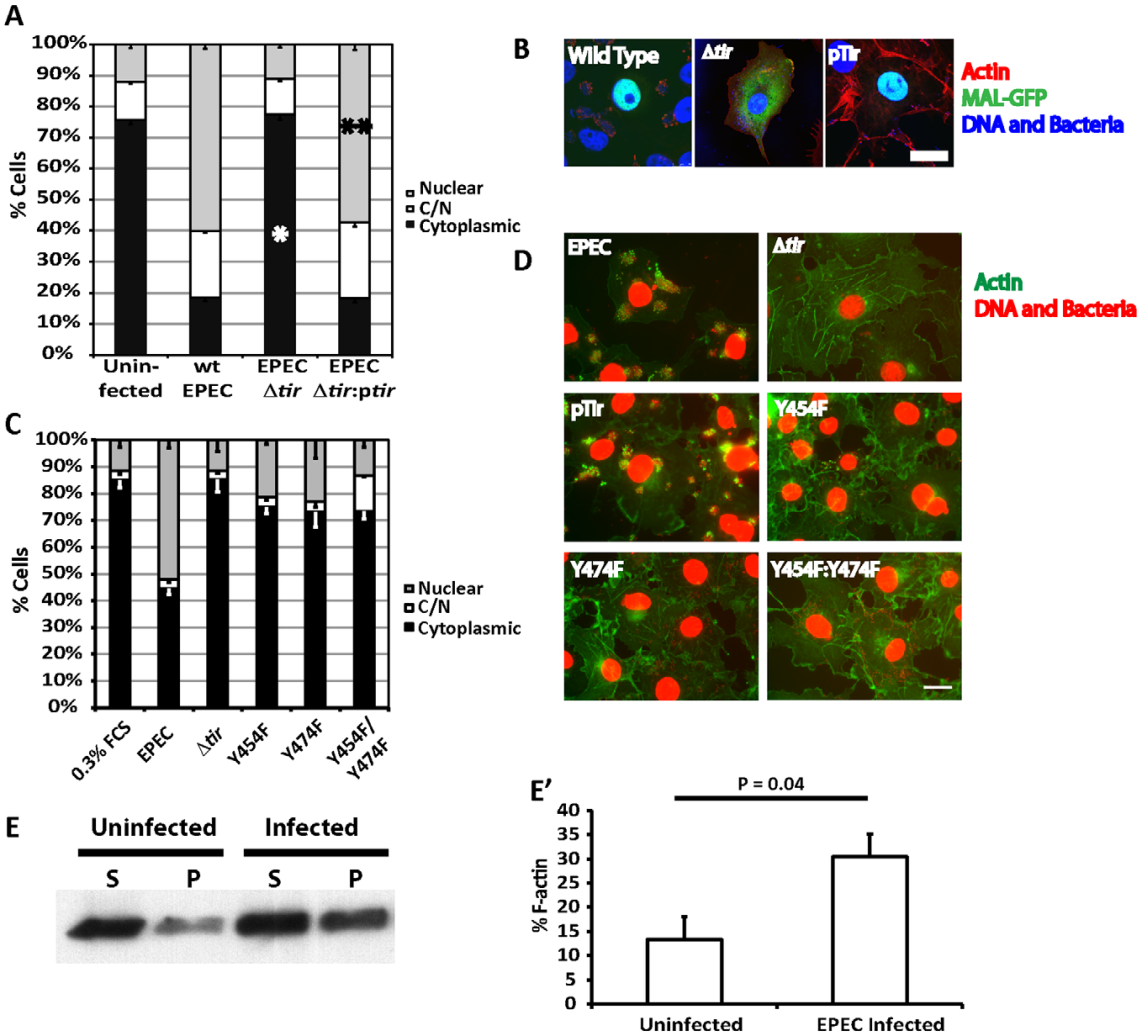
Tir is essential for EPEC induced MAL-GFP translocation. A. EPEC Δ*tir* cannot induce the nuclear translocation of MAL-GFP. COS-7 cells were transfected with MAL-GFP, serum starved then infected with the indicated bacteria. The percentage of transfected cells that had MAL-GFP in the nucleus, cytoplasm or both nucleus and cytoplasm was determined. Data are the means of at least 3 experiments where a minimum of 150 transfected cells were counted for each condition of each experiment. Values are means ± standard deviation. Relative to the uninfected control *P = <0.45, ** P = 7.27^−7^. B. Representative images from A, MAL-GFP- (green), F-actin (red), DNA/bacteria (blue). C. Phosphorylation of Tir residues Y454 and Y474 is necessary for EPEC-induced MAL-GFP translocation. D. Representative images from C, F-actin (green), DNA/bacteria (Red). Scale Bar = 20 µm. E. Cells untreated or infected with EPEC for 3.5 hours were lysed and separated into 100,000 –g supernatant (S) or pellet (P) fractions. Equal amounts were separated by SDS-PAGE and actin in each fraction detected using an anti-actin antibody. E′ F-∶G-actin ratios from E were quantified as described in materials and methods. The mean % F-actin from at least 3 experiments is shown ± standard deviation.

Given the relationship between SRF and actin, and the actin cytoskeleton rearrangements induced by pedestal formation, we hypothesized that pedestal formation would be necessary to induce the observed nuclear accumulation of MAL-GFP. To test this hypothesis we infected COS-7 cells with EPEC Δ*tir*, which are unable to build actin pedestals [17]. In COS-7 cells infected with EPEC Δ*tir*, MAL-GFP remained predominantly cytosolic, with 77.5% of cells±1.75%, displaying no significant difference to the 0.3% FCS control 75.7%±1.46% (Figure 4A and B). To confirm that this loss of phenotype was due solely to the lack of Tir, we infected COS-7 cells with EPEC Δ*tir* rescued with a plasmid carrying Tir (pTir [17]). COS-7 infected with EPEC Δ*tir*/pTIR efficiently rescued the MAL-GFP nuclear accumulation phenotype, with 57.38%±1.73% of cells exhibiting nuclear localization of MAL-GFP (Figure 4A and B). Similar to COS-7 cells infected with wild-type EPEC (60.08%±1.03% nuclear). Therefore the formation of the F-actin-rich pedestal is clearly necessary for EPEC induced MAL-GFP accumulation in the nucleus. This is supported by the fact that no SRF target genes were induced by infection with EPEC Δ*tir* (Figure 3).

Among all the secreted EPEC effector proteins, only Tir is involved in signalling host cells to generate actin pedestals [18]. Phosphorylated Tir Y474 binds the adapter protein Nck to recruit N-WASP [17], while phosphorylated Y454 stimulates a lower efficiency Nck-independent pathway [19].

To determine if the activation of SRF by pedestal formation was dependent on a specific pathway, i.e. Nck dependent or independent, we infected MAL-GFP expressing COS-7 cells with EPEC Δ*tir* strains rescued with pTIR Y474F, Y454F or Y474F/Y454F mutants and determined the percentage of cells displaying cytosolic or nuclear localization of MAL-GFP. Infecting cells with EPEC Δ*tir* expressing either TirY454F or TirY474F significantly reduced the percentage of cells showing nuclear accumulation of MAL-GFP to 21.2%±0.6% (p = 3.96*10^−5^) and 23.04%±6.8% (p = 2.9*10^−5^) respectively, relative to those infected with wild-type EPEC (51.9%±2.9%, Figure 4C and D). The double mutant decreased the percentage of cells with nuclear MAL-GFP further, to a level not significantly different from the EPEC Δ*tir* control, 14.86%±1.26% and 11.39%±1.7% respectively, which suggests that stimulation of actin assembly by Tir is crucial for MAL-GFP nuclear accumulation in response to EPEC infection.

To further understand the Tir requirements for EPEC induced MAL-GFP translocation we expressed a plasma membrane-targeted construct containing the intimin-binding extracellular loop and the COOH-terminal cytoplasmic domain of Tir (TirMC), or a similar plasma membrane-targeted Tir construct in which Tyr474 had been mutated to phenylalanine (TirMC (Y474F)) [18]. These constructs were clustered at the plasma membrane by infecting cells with EPEC Δ*tir*, which cannot induce MAL-GFP translocation. Clustering the COOH terminus of Tir beneath the plasma membrane is sufficient to drive actin pedestal formation [18]. TirMC or TirMC (Y474F) expressing cells were identified by anti-HA fluorescence. 68.2%±6.17% of cells expressing TirMC displayed a nuclear localization of MAL-GFP after 5 hours of infection with EPEC Δ*tir* (Figure S2). In contrast, nuclear localization of MAL-GFP was significantly reduced to 27.9%±4.03% (p = 0.000225) in cells expressing TirMC (Y474F) following infection with EPEC Δ*tir* (Figure S2). These results suggest that the pathway of activation is unimportant, but rather the act of building and maintaining the pedestal is necessary to activate SRF.

### EPEC infection in epithelial cells alters F∶G-actin ratios

To test the hypothesis that EPEC-induced nuclear accumulation of MAL is driven by infection-driven changes in G∶F-actin ratios within the host cell, we quantified the G- and F-actin in EPEC infected cells relative to uninfected cells. Cells were extracted with a Triton X-100 lysis buffer (see materials and methods) and separated into 100,000-g supernatant and pellet fractions. Under these conditions G-actin is found in the supernatant and F-actin in the pellet. As shown in figure 4E, at timepoints early in the infection, consistent with the kinetics of pedestal formation in tissue culture cells, we could detect an average 2.3-fold increase in F-actin in EPEC infected cells (Figure 4E).

Together these data demonstrate that pedestal formation can alter G∶F-actin ratios in infected cells and that pedestal formation is necessary for accumulation of MAL-GFP in the nucleus.

### EPEC and EHEC Tir components are interchangeable for infection-induced MAL-GFP nuclear accumulation

**Figure 5:**
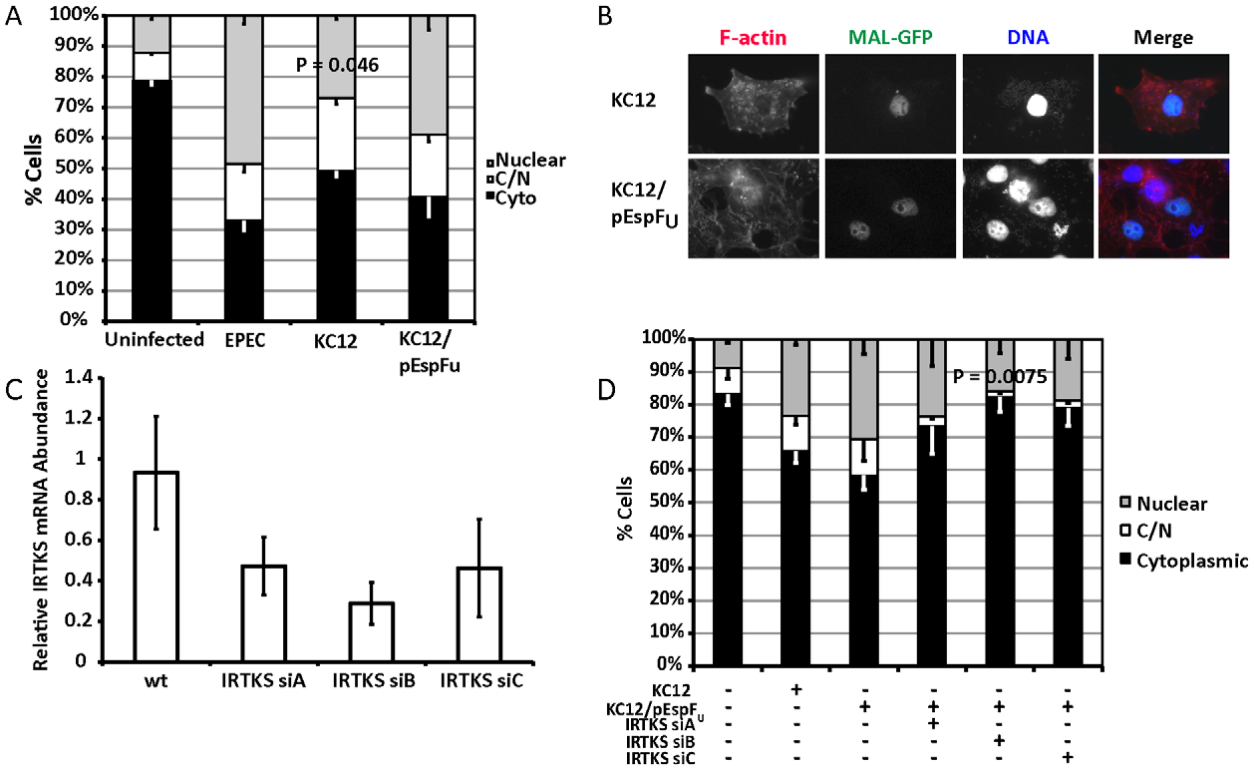
Tir^EHEC^ can rescue the EPEC Δ*tir* loss of MAL-GFP translocation. A. Cellular distribution of MAL-GFP in serum-starved COS-7 cells infected with bacteria as indicated. Data are the means of at least 3 experiments where a minimum of 150 transfected cells were counted for each condition of each experiment. Values are means ± standard deviation. B. Representative images of pedestal formation induced by Tir^EHEC^ rescue strains. C. COS-7 cells transfected with siRNA targeting IRTKS. After 72 hours knockdown efficiency was assessed using real-time quantitative RT-PCR with IRTKS specific primers and normalized to *GAPDH*. Shown are means of three experiments ± standard deviation. D. Cellular distribution of MAL-GFP in IRTKS knockdown cells infected with KC12 pEspF_U_. Data are means of three experiments ± standard deviation. Scale bar = 20 µm.

EPEC and EHEC induce attaching and effacing lesions by different signalling mechanisms. Whereas EPEC Tir is the only translocated EPEC effector required to trigger pedestal formation, EHEC translocates two effectors, Tir(EHEC) and EspFu (also known as Tccp) to generate pedestals in an Nck-independent manner [18], [20]. We reasoned that if the act of building pedestals was enough to activate SRF, then Tir^EHEC^+EspF_U_ would be commensurable to Tir^EPEC^ in inducing MAL-GFP nuclear accumulation. As such, we tested to see if Tir^EHEC^ could rescue the EPEC Δ*tir* phenotype. COS-7 cells expressing MAL-GFP were infected with EPEC Δ*tir* exogenously expressing Tir^EHEC^ (KC12) or Tir^EHEC^+EspF_U_ (KC12/pEspF_U_) [21]. Post-infection, the cells were fixed, stained and MAL-GFP localization determined by fluorescence microscopy. Under these conditions 39.9%±4.56% of cells infected with KC12/pEspF_U_ exhibited a nuclear localization of MAL-GFP compared to 26.9±2.06% of cells infected with KC12 (Figure 5A and B). The nuclear localization of MAL-GFP induced by KC12 was significantly reduced compared to cells infected with wild-type EPEC (48.4%±4.6%).

Recent studies have demonstrated that the I-BAR family protein insulin receptor tyrosine kinase substrate (IRTKS) is central to EHEC pedestal formation, forming a ternary complex with Tir^EHEC^, pEspF_U_ and N-WASP necessary for pedestal formation [22], [23]. We tested whether IRTKS was necessary for MAL-GFP translocation in the Tir^EHEC^ rescue system. We first confirmed the ability of three siRNAs to knockdown IRTKS in COS-7 cells. siRNA B reproducibly gave the best knockdown (Figure 5C). We tested the ability of EPEC KC12/pEspF_U_ to induce nuclear accumulation of MAL-GFP in the IRTKS knockdown cells. Knockdown of IRTKS significantly reduced the ability of KC12/pEspF_U_ to induce nuclear accumulation of MAL-GFP from 30.5% of cells in the control to 15.9%±1.79% in knockdown cells (Figure 5D).

These data are consistent with significant actin-rearrangement induced by pedestal formation, being central to the nuclear accumulation of MAL-GFP. KC12 are inefficient builders of actin pedestals [21] and, under these conditions, cause very little nuclear translocation of MAL-GFP. However, the rescue expressing Tir^EHEC^ and EspFu, the two EHEC effectors required for robust pedestal formation, induces nuclear accumulation of MAL-GFP comparable to wild type EPEC. Secondly IRTKS has been shown to be necessary for efficient pedestal formation by EHEC [22], [23]. In the Tir^EHEC^ rescue system used here, knockdown of IRTKS led to reduced pedestal formation and a subsequent lack of MAL-GFP nuclear accumulation and activation of SRF.

### ABRA and FLRT3 are SRF activators required for EPEC-induced MAL-GFP translocation

**Figure 6:**
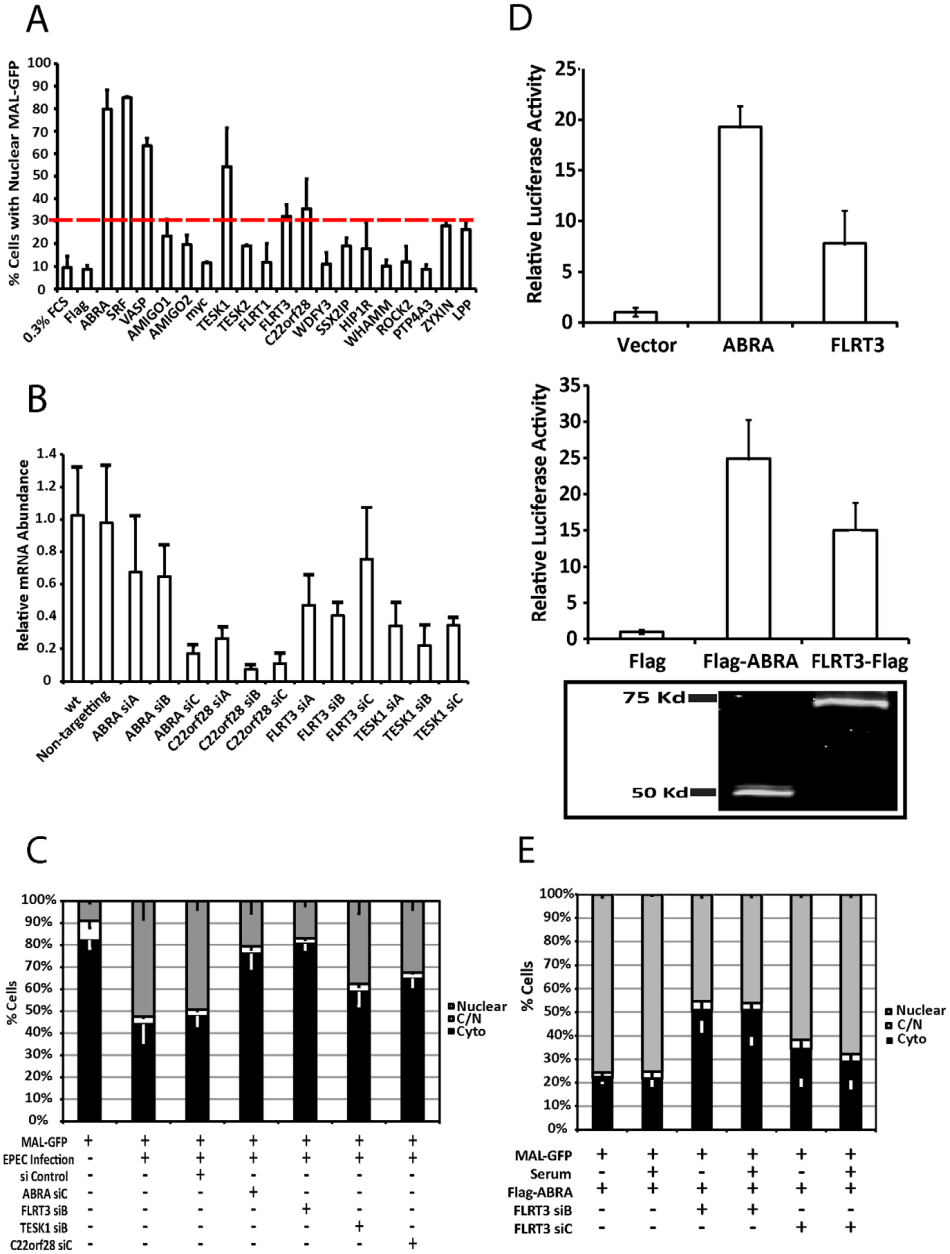
ABRA and FLRT3 are SRF activators required for EPEC-induced MAL-GFP translocation. A. The percentage of transfected cells displaying nuclear localization of MAL-GFP after serum starvation. COS-7 cells were cotransfected with MAL-GFP and cDNA expression constructs as indicated. After 18 hours they were serum starved for 24 hours then fixed and stained. The dotted red line represents the cut-off threshold for the percentage of cells displaying nuclear MAL-GFP required for a gene to be declared a hit. The cut-off (30%) was defined as a 2-fold increase over background (10% for 0.3% FCS or empty vector controls). Data represents the mean of 500 cells from 3 individual experiments ± standard deviation. B. COS-7 cells transfected with siRNA targeting the indicated genes or a non-targeting siRNA. After 72 hours knockdown efficiency was assessed using real-time quantitative RT-PCR with FLRT3, TESK1 or C22orf28 specific primers and normalized to GAPDH. Shown are means of three experiments, each using independent cDNA samples, ± standard deviation. C. EPEC-induced nuclear translocation of MAL-GFP is significantly reduced in ABRA (P = 0.0021) and FLRT (P = 0.000363) knockdown cells. Localization of MAL-GFP in serum starved COS-7 cells with respective protein knockdown after infection with EPEC. Data are the means of three experiments ± standard deviation. D. ABRA and FLRT3 induce transcription of SRE-luciferase. E. FLRT3 knockdown significantly reduces ABRA-induced nuclear translocation of MAL-GFP, P = 0.0017 relative to the no siRNA control. Data are the means of three experiments ± standard deviation.

To identify host factors involved in the nuclear translocation of MAL-GFP we tested the ability of a number of known or putative actin binding proteins to induce nuclear translocation of MAL-GFP. Candidate expression plasmids were cotransfected with MAL-GFP into COS-7 cells and the cellular localization of MAL-GFP was determined by fluorescence microscopy. We defined the minimum cut-off point for activation as a 2-fold increase over the vector only control. Both ABRA and SRF were able to significantly induce the nuclear accumulation of MAL-GFP (Figures 6a and S3). 79.9%±6.04% of cells overexpressing ABRA exhibited nuclear localization of MAL-GFP and 87.05%±1.14% of cells overexpressing SRF displayed nuclear localization of MAL-GFP, an 8-fold increase over the vector only control. In addition we, identified three novel genes that could induce nuclear accumulation of MAL-GFP by overexpression. These genes are FLRT3 (32.2% nuclear), TESK1 (54.3% nuclear) and C22orf 28 (35.59% nuclear, Figures 6A and S3).

ABRA is an actin binding protein that can induce nuclear accumulation of MAL-GFP and activate SRF [24]. C22orf28 (also known as HSPC117 or FAAP in mice) is a cell adhesion protein with Ankyrin repeats, that interacts with vinculin and talin [25]. TESK1 (testis-specific kinase 1) is a LIM kinase-related serine/threonine kinase that has been shown to influence actin organization via its ability to phosphorylate cofillin [26]. FLRT3 (Fibronectin leucine rich transmembrane protein 3) is a putative type I transmembrane protein containing 10 leucine-rich repeats, a fibronectin type III domain, and an intracellular tail. It has been implicated in neurite outgrowth [27] and cell adhesion [28] and has a predicted SRF binding site in its promoter. Furthermore we have recently demonstrated that Flrt3 is induced by bacterial infection [29].

To assess the significance of the overexpression screen hits for EPEC induced activation of SRF, we determined the ability of EPEC to induce MAL-GFP translocation in the absence of each protein individually. Knockdown efficiency was first established for the three candidate genes FLRT3, TESK1 and C22orf28 (Figure 6B). While C22orf28 and TESK1 had no effect, knockdown of ABRA or FLRT3 significantly reduced MAL-GFP nuclear translocation induced by EPEC to 20.6%±3.2% and 16.9±11.16% respectively (Figure 6C). This indicates that ABRA and FLRT3 are both required for EPEC-induced translocation of MAL-GFP to the nucleus. Furthermore we confirmed the ability of ABRA and FLRT3 to activate an SRE-luciferase reporter in the absence of serum (Figure 6D). Both epitope tagged and untagged constructs of ABRA and FLRT could induce transcriptional activity of a luciferase gene under the control of the serum response element. Together these results demonstrate that ABRA and FLRT3 are components of the pathway involved in EPEC induced signaling to SRF.

### ABRA-induced GFP-MAL translocation is dependent on FLRT3

As both ABRA and FLRT3 can induce nuclear accumulation of MAL-GFP and are required for EPEC-induced nuclear accumulation of MAL-GFP (Figures 6), we sought to undertake an epistasis analysis of ABRA and FLRT3. We tested to see if FLRT3 knockdown would inhibit ABRA-induced translocation of MAL-GFP. We found that ABRA-induced nuclear accumulation of MAL-GFP was significantly reduced to 45.4%±7.6% of cells in the FLRT3 knockdown cells compared to 75.7%±3.3% in the wild type control (Figure 6E and S2C). In the reciprocal experiment, ABRA knockdown had no effect on FLRT3 induced nuclear accumulation of MAL-GFP (Figure S4). These findings are consistent with FLRT3 functioning downstream of ABRA but upstream of MAL.

Surprisingly FLRT3 siRNA reduces ABRA-induced MAL nuclear localization with or without serum induction under these conditions, whereas FLRT3 siRNA alone has no effect on serum induced MAL nuclear localization (Figure S2C). It therefore appears that the combination of ABRA overexpression and FLRT3 knockdown can block serum induction of MAL.

### FRT3 and ABRA localize to the EPEC pedestal and ABRA is necessary for maintenance of discreet pedestals

**Figure 7:**
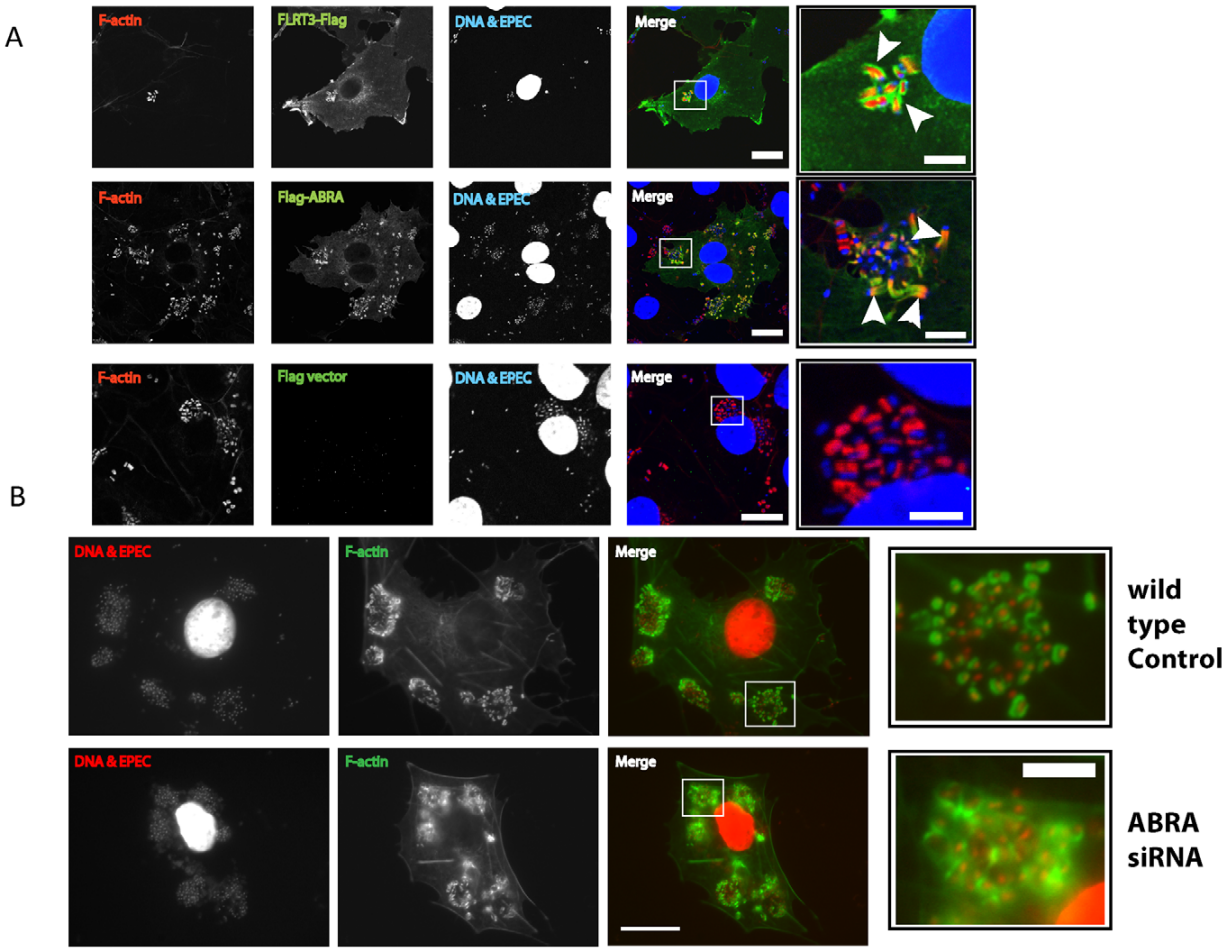
ABRA is necessary for correct pedestal formation. A. COS-7 cells transfected with empty pCMV-3xFlag vector, FLRT3-Flag or Flag-ABRA. Cells were infected with EPEC, fixed and stained with an anti-Flag antibody. FLRT3-Flag and Flag-ABRA are enriched at the EPEC pedestal (Arrowheads). B. Wild-type and ABRA knockdown cells were infected with EPEC for a total of 5 hours, fixed and stained with DAPI (red) and phalloidin (green). Pedestals in ABRA knockdown cells are disorganised (close-up). Scale bars = 20 µm and 5 µm in close-up panels.

We next sought to establish the cellular localization of each of the candidate proteins during EPEC infection to determine their involvement in pedestal formation (Figure 7 and S5). ABRA colocalized with F-actin and was enriched in the EPEC pedestal (Figure 7A, arrowheads), while SRF was always localized to the nucleus (Figure S3). FLRT3 localized to the plasma membrane and was enriched at pedestal sites (Figure 7A).

With ABRA localizing to the EPEC pedestal and knockdown inhibiting MAL-GFP nuclear accumulation, we questioned whether loss of ABRA would also affect EPEC pedestal morphology. Although pedestals associated with single bacteria appeared normal, pedestals associated with micro colonies of EPEC appeared unstructured (Figure 7B and S6), taking on an appearance akin to a ruffle. This suggests that maintenance of discreet pedestals is lost in the ABRA knockdown, with single pedestals merging into one large structure. We therefore suggest that ABRA is necessary for proper pedestal formation, which in turn, is necessary for SRF activation.

## Discussion

Recent studies have suggested a connection between pathogen mediated actin re-organization and serum response factor (SRF) transcriptional programs [11]. We screened a panel of gastrointestinal tract-associated pathogens for the ability to induce nuclear accumulation of MAL-GFP. Surprisingly only EPEC caused a significant change in MAL-GFP localization under the infection conditions tested. We suspected *S.* Typhimurium would have some effect on MAL-GFP localization. It has been shown that *S.* Typhimurium induces actin ruffles during entry and activates host cell Rho-GTPases [30], [31]. However, unlike EPEC infection, *Salmonellae* rapidly return the host cell cytoskeleton to its resting state following engulfment, via the action of the effector protein SptP [32]. Perhaps this down-modulation of actin polymerization by *S.* Typhimurium is sufficient to stifle the activation of SRF, whereas the prolonged actin remodelling induced by EPEC infection is not.

We confirmed that MAL translocation correlates with upregulation of SRF target genes during EPEC infection (Figure 3 and S1). EPEC infection selectively activates SRF target genes, most significantly EGR2 and IL-6, but also CDC42EP3, ARHGDIB, ACTA2 and VAV3, relative to uninfected controls. None of these genes were activated by EPEC Δ*tir* infection. CDC42EP3, ARHGDIB, and VAV3 are all involved in Rho, Rac or Cdc42-mediated signalling and are consistent with the Rho dependent pathway of MAL translocation and SRF activation [7]. Whether upregulation of these genes is required for pedestal formation or pathogen survival, or is a natural consequence of pedestal formation is unclear at this time, but warrants further study.

EGR2 is an immediate-early, zinc finger transcription factor with two serum response elements in its 5′ flanking sequence [33]. EGR2 can be activated by a number of infectious agents including viruses (Human T-cell Leukemia virus type 1), bacteria, and parasites (*Toxoplasma gondii*) [34]–[36]. Interestingly in *T. gondii* infection EGR2 induction was dependent on rhoptry secretion, a process analogous to secretion of proteins into a host cell by the bacterial type III secretion system [36]. Likewise, we find the secreted protein Tir to be essential for EPEC-induced activation of EGR2. In other infections EGR2 expression is often accompanied by EGR1 and c-FOS. Under our experimental conditions the expression of EGR1 and c-FOS was not induced. This may suggest that this is an EPEC-specific response rather than a general innate pathogen response.

It is clear that host signalling pathways are activated in response to many infectious agents, suggesting they are functioning in innate immunity. Although IL-6 is a well-known SRF target [10], [37] its expression can be induced by a number of bacteria [38], it is possible therefore, that IL-6 may function as an innate sentinel in this context. The fact that none of these genes were induced by infection with EPEC Δ*tir* demonstrates that pedestal formation is fundamental to this signalling cascade.

Tir is an essential effector for the assembly of F-actin pedestals. Following secretion, Tir inserts into the host cell membrane, presenting an extracellular domain that binds the bacterial surface protein intimin [39]. The C-terminal region of Tir^EHEC^ is phosphorylated at Tyr474 by host-cell kinases [40] in a manner similar to host receptor phosphorylation [41], [42]. Phosphorylated Y474 and its flanking residues bind Nck via its SH2 domain [17], [43]. Nck subsequently recruits and activates N-WASP stimulating ARP2/3 driven F-actin assembly. In addition, Tir^EPEC^ can promote weak actin polymerization in an Nck-independent manner via phosphorylation of Tir residue Y454 [19]. In this report we show that Tir is essential for EPEC-induced MAL-GFP nuclear accumulation and subsequent transcriptional activation of selective SRF target genes. Infection of epithelial cells with EPEC Δ*tir* does not induce MAL-GFP nuclear accumulation, but this phenotype is rescued by the exogenous expression of Tir (Figure 4A). This is consistent with actin rearrangement driven by pedestal formation being key for SRF activation rather than a translocated effector activating SRF directly. In further support of this idea Tir^EHEC^+pEspF_U_ could also rescue the EPEC Δ*tir* phenotype (Figure 5). Tir^EHEC^ is functionally divergent from Tir^EPEC^ [17], [44], [45]. Tir^EHEC^ lacks a residue equivalent to Tyr474 [40], is not tyrosine phosphorylated in cells [46] and does not bind Nck [43]. To efficiently form actin pedestals EHEC requires a second translocated effector EspF_U_ (TccP) [20], [21]. EspF_U_ is recruited indirectly to Tir by IRTKS [22], [23], where it can than activate N-WASP which results in actin polymerization. Although the initial signalling methods used to recruit and activate host cell nucleation factors between the related pathogens are different, the net result is the same. Likewise, single mutations of either Tir^EPEC^ Y454 or Y474 to non-phosphorylatable phenylalanines drastically reduced the nuclear accumulation of MAL-GFP to similar levels (Figures 4C and S2), suggesting that Nck dependent or independent activation of N-WASP is irrelevant to EPEC-induced MAL-GFP nuclear accumulation. In addition, knockdown of SRF reduced EPEC-induced MAL-GFP accumulation in the nucleus to near uninfected levels (Figure 2). This is likely the result of altered cytoskeletal gene expression, resulting from the loss of SRF.

In order to identify the host signaling cascades that are co-opted by bacterial virulence factors to regulate the cytoskeleton, we sought a scheme to identify genes generally employed in mammalian cytoskeleton control. We picked known and putative actin-associated or regulatory genes and tested their ability to induce nuclear accumulation of MAL-GFP. Novel genes inducing MAL-GFP nuclear accumulation with probable involvement in actin-cytoskeletal rearrangement were then evaluated for involvement in host-pathogen interactions.

We identified FLRT3, TESK1 and C22orf28 as novel inducers of MAL nuclear accumulation and confirmed the involvement of FLRT3 in EPEC induced MAL translocation by siRNA (Figure 6). Overexpression of ABRA induced nuclear accumulation of MAL-GFP consistent with published data for the Murine homologue STARS [14], [24]. Knockdown of ABRA significantly decreased EPEC induced accumulation of MAL-GFP in the nucleus, suggesting that ABRA is a necessary component in the signaling pathway. In addition we found ABRA was enriched in EPEC pedestals and that ABRA knockdown adversely affected pedestal morphology. STARS has been shown to activate SRF and stabilize the F-actin cytoskeleton in a RhoA dependent manner, with the carboxy terminal being sufficient and necessary to activate SRF and bind actin [24]. The pedestal phenotype observed in ABRA knockdown cells is consistent with ABRA stabilizing the F-actin cytoskeleton in this context (Figure 7B). Loss of this stabilization function in microcolonies leads to the dissolution of discreet pedestals and results in a structure more similar to a ruffle.

Under our experimental conditions overexpression of SRF also resulted in the nuclear accumulation of MAL-GFP. The specific reason for this is currently unclear. Currently the prevailing hypothesis states that MAL continually shuttles between the cytoplasm and the nucleus. Perhaps MAL has a higher binding affinity for SRF than G-actin, and upon entering the nucleus, preferentially complexes with SRF and is retained in the nucleus.

Transcription of SRF is controlled by SRF its self [47], the overexpression of SRF may be interpreted by the cell as activation of the pathway, leading to an upregulation of SRF target genes and subsequent decrease in G-actin. These are just two potential hypotheses that may not be mutually exclusive, but warrant further study.

**Figure 8:**
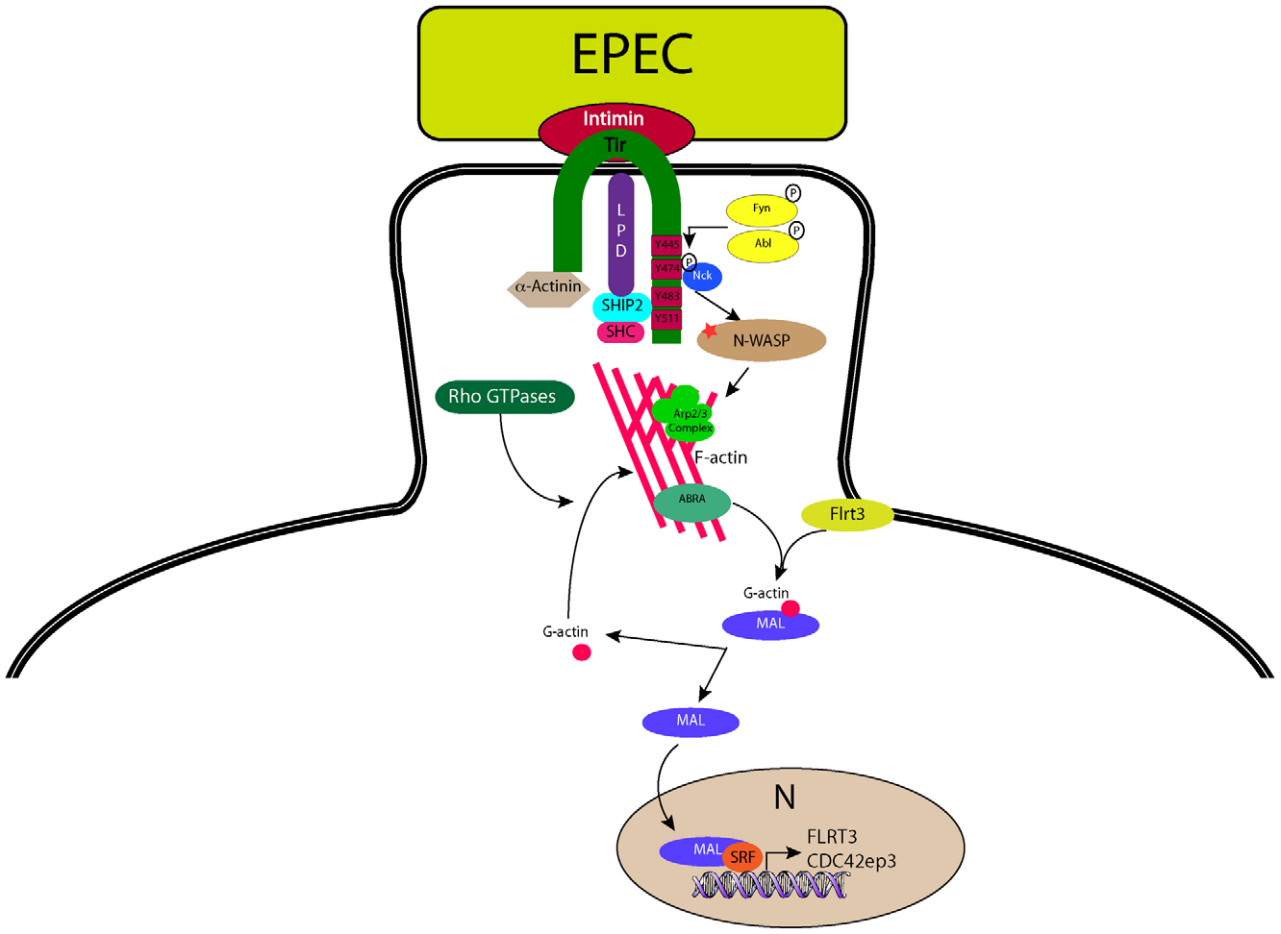
A model for EPEC-induced activation of host-cell transcription factor SRF. During infection with the extracellular pathogen EPEC, Tir translocates to the host cell and inserts into the plasma membrane where it interacts with the bacterial surface protein intimin and anchors it to the cell. The C-terminus of Tir is phosphorylated by host cell kinases leading to the recruitment and binding of Nck. N-WASP and the Arp2/3 complex are recruited leading to the generation of actin filaments beneath the bacterium. ABRA can bind actin in the newly formed (or forming) pedestal and stabilise the structure. The change in G-actin to F-actin ratio induced by pedestal formation is “sensed” by MAL via direct or indirect actions from ABRA and FLRT3, whereupon it is freed from its inhibitory complex with G-actin, to enter the nucleus and interact with SRF, driving transcription of target genes.

Of the proteins identified in this study ABRA and FLRT3 localized to the EPEC pedestal (Figure 7), and were both necessary for EPEC-induced translocation of MAL-GFP (Figure 6). Epistasis analysis showed that knockdown of FLRT3 could significantly reduce ABRA-induced nuclear accumulation of MAL-GFP, but ABRA knockdown had no effect on FLRT3-induced nuclear accumulation of MAL-GFP (Figure 6E and S4). This places FLRT3 downstream of ABRA and identifies it as an intermediary protein from pedestal to nucleus. Based on this data we hypothesize a new model (Figure 8), where EPEC-induced remodeling of the actin cytoskeleton, via Tir, activates SRF in an ABRA and FLRT3 dependent manner. Our findings therefore reveal a novel mechanism for pathogen-induced activation of a host transcription factor. They shed light on the relationship between ABRA and SRF and identify FLRT3 as a new component of this signalling pathway.

## Materials and Methods

### Cell culture and bacterial strains

COS-7 cells were obtained from ATCC and routinely cultured in DMEM supplemented with 10% iron supplemented foetal calf serum (Hyclone, USA) and 40 µg/ml gentamycin sulphate. EPEC strains carrying *tir* deletions and complementation plasmids have been described previously [17]. *S.* Typhimurium SL1344 DsRed2 was given by Dr. H.C. Reinecker.

### Constructs

MAL was amplified from a mouse cDNA template by PCR using a forward primer introducing a XhoI site: 5′ CTCGAGATGCCGCCTTTGAAAAGCCCC 3′; and a reverse primer introducing a SacII site: 5′ CCGCGGCAAGCAGGAATCCCAGTGGAG 3′. The resulting product was ligated into pEGFP-N1 (Clontech).

To generate constructs for mammalian expression of ABRA, SRF, FLRT3, TESK1and C22orf28 the coding sequences were amplified from cDNA clones in pCMV-SPORT6 obtained from Open Biosystems. Coding sequences were amplified using the primers in Table 1.

**Table 1:** Cloning primers.

Gene	Forward Primer	Reverse Primer
ABRA	ACATCAT**GAATTC**AAGCTCCGGGCGAAAAGGAAAGT	ACATCAT**GCGGCCGC**TCACTTGAGTAGCGTAATCACAACATGGTC
SRF	CGCGGG**GAATTC**GTTTACCGACCCAAGCTGGGGCCGCGGCGGCTCT	GGCCGAT**gcggccgc**TCATTCACTCTTGGTGCTGTGGGCGGTGTCCAGGTTCA
FLRT3	cgcggg**AGATCT**gatATCAGCGCAGCCTGGAGCATCTTCCTCATCGGGA	GGCCGAT**gcggccgc**TCATGAGTGTGAGTGATCTGAGTCTGGAATACCAC
TESK1	cgcggg**GAATTC**gtGCCGGGGAACGGCCCCCACTGCGGGGCCCTGG	GGCCGAT**gcggccgc**CTAAGAGCGTGCCCCAGGCAGCTGCAGGCTG
C22ORF28	cgcggg**GAATTC**gtAGTCGCAGCTATAATGATGAGCTGCAGTTCTTG	GGCCGAT**gcggccgc**CTATCCTTTGATCACAGCAATTGGTCTCAGTTTA

Introduced restriction sites are highlighted in bold.

After digestion with the appropriate restriction enzymes, the coding sequence was subcloned into N-terminally tagged pCMV-3xFlag or -3xMyc vectors derived from the pCMV-Myc vector (Clontech, catalog no. 631604).

### Transfections and knockdowns

COS-7 cells were plated onto 18 mm glass coverslips in 12-well plates at a density of 4×10^4^ cells per well. After 24 h cells were transfected in antibiotic-free medium with MAL-GFP plus additional myc- or Flag- tagged constructs (in a modified pCMV vector, Clontech, USA), where noted, at a 1∶1 ratio, using GeneJuice (Novagen, UK), according to the manufacturers instructions. 18 h post-transfection cells were washed twice in PBS and incubated in DMEM 0.3% FCS for a further 18 h, prior to bacterial infections.

### RNA interference

COS-7 cells were plated onto 18 mm glass coverslips in 12-well plates at a density of 4×10^4^ cells per well. After 24 h, 20 pmol of modified RNA oligoduplexes (Stealth RNAi; Invitrogen), were transfected into each well using X-tremeGENE (Roche), according to the manufacturers instructions. siRNA silencing sequences are shown in Table 2.

**Table 2:** siRNA silencing sequences.

Gene	Sense	Antisense
ABRA siA	UCUCUUUGACAGAUACGUUCGUAUU	AAUACGAACGUAUCUGUCAAAGAGA
ABRA siB	UCAGAUAAAGUAGUGGGCAUUCUCA	UCAGAAUGCCCACUACUUUAUCUGA
ABRA siC	CCAACCUGGUGUCUGAGCUAACCAA	UUGGUUAGCUCAGACACCAGGUUGG
SRF	GCUACACGACCUUCAGCAAGAGGA	UUCCUCUUGCUGAAGGUCGUGUAG
IRTKS siA	GGCGCUUCUGCUUUCUGGUUGAUAA	GGCGCUUCUGCUUUCUGGUUGAUAA
IRTKS siB	CCCGACUACUUGGAAUGCUUGUCCA	UGGACAAGCAUUCCAAGUAGUCGGG
IRTKS siC	CCCGAAUUCACAAAGGGUAAAUAAU	AUUAUUUACCCUUUGUGAAUUCGGG
TESK1 siA	CAAGAACUGUCUAGUCCGACGGGAA	UUCCCGUCGGACUAGACAGUUCUUG
TESK1 siB	CCUAGAUCAGGACCCGUCCUCAAUA	UAUUGAGGACGGGUCCUGAUCUAGG
TESK1 siC	ACUUUGGCCUGGAUGUGCCUGCUUU	AAAGCAGGCACAUCCAGGCCAAAGU
C22ORF28 siA	CAAUGAUCGGCAGUUGGCUUGUGCU	AGCACAAGCCAACUGCCGAUCAUUG
C22ORF28 siB	UAGUUAUGUUCUUACUGGCACUGAA	UUCAGUGCCAGUAAGAACAUAACUA
C22ORF28 siC	CGUGUUGCCUCACCCAAACUGGUUA	UAACCAGUUUGGGUGAGGCAACACG
FLRT3 siA	CCCGCAUUUGGAUCUAUAACAGAA	UUCUGUUAUAGAUCCAAAUGCCGGG
FLRT3 siB	CCCUAUCUGGAAGAAUUACAUUUAG	CUAAAUGUAAUUCUUCCAGAUAGGG
FLRT3 siC	UCAACCUAGUUAAUUUGACAGAGCU	AGCUCUGUCAAAUUAACUAGGUUGA

Cells were serum starved 48 h post-transfection as described above and infected, fixed and stained as described below.

### Reverse transcription and real-time PCR

RNA extraction was performed by using an RNeasy kit (Qiagen) in accordance with the manufacturer's instructions. 500 ng of total RNA was reverse-transcribed using an iScript cDNA synthesis kit (Bio-Rad). The gene expression reported is representative of three independent experiments. Real-time quantitative PCR was performed in triplicate in a Bio-Rad iCycler thermal cycler equipped with an iQ5 optical module using the iQ SYBR Green Super Mix (Bio-Rad). In brief, 100 ng of reverse-transcribed cDNA was used for each PCR with forward and reverse primers at 250 nM. The thermal cycling conditions were 4 min at 95°C, followed by 40 cycles at 94°C for 15 s and 59°C for 1 min. Values were normalized to that of *GAPDH*. All PCR products were analyzed on a 2% agarose gel to verify the correct size of the amplicons. RT-PCR primer sequences are shown in Table 3.

**Table 3:** RT-PCR primer sequences.

Gene	Forward Primer	Reverse Primer
ABRA	CCAATCACACCCCCTACTTCA	CCGTTTTGGACACCTCTTTC
SRF	CAAGATGGAGTTCATCGACAACA	CGAGTTGAGGCAGGTCTGAAT
IRTKS	AGAGCACCTACCGGAATGTTA	TGGCAATCTCACCGATCTTGG
TESK1	GGGCAACACACTACGGGAAG	GGTCGCGGTGAAATACACCTT
FLRT3	ATGAATTTCCTACCAACCTCCCA	AGTTGCTGTCTCGGAATGCTC
C22orf28	GCTGGAGGATCAAGAAGGGC	CCATGTTCCCAATAGCAAACCC
GAPDH	TCATCTCTGCCCCCTCTGCTGA	CGCCAGTAGAGGCAGGGATGATG
CFL1	TTCAACGACATGAAGGTGCGT	TCCTCCAGGATGATGTTCTTCT
VAV3	GCGCACTCCATCAACCTGAA	TCCAAACGTCTCACAACAGGC
RSU1	ACCGTCTTTTCAAATGGCCTG	GCCAGAAGTTTAGACCTTGCTCT
ARHGDIB	GTGGTGACAGATCCGAAAGCC	CTGTAGGTGTGCTGAACGTATT
FYN	TCTGCTGCCGCCTAGTAGTT	ACAGACAGATCGGTAAGCCTT
VCL	TCTCCCACCTGGTGATAATGC	TGGTTTGAACAGTCTCTTTTCCA
CYR61	CTCGCCTTAGTCGTCACCC	CGCCGAAGTTGCATTCCAG
ACTA2	CAGGGCTGTTTTCCCATCCAT	GCCATGTTCTATCGGGTACTT
EGR1	ACCTGACCGCAGAGTCTTTTC	GCCAGTATAGGTGATGGGGG
EGR2	ATCCCAGTAACTCTCAGTGGTT	CTCCACCGGGTAGATGTTGT
FOS	CGGGCTTCAACGCAGACTA	GGTCCGTGCAGAAGTCCTG
c-FOS	CGGGCTTCAACGCAGACTA	GGTCCGTGCAGAAGTCCTG
CDC42ep3	AAGACCCCAATTTACCTGAAAGC	TGGCGAAAGTCTCCAAGCG
IL-6	AAATTCGGTACATCCTCGACGG	GGAAGGTTCAGGTTGTTTTCTGC

### Bacterial infection conditions

For EPEC, AIEC and *E.coli* K12 infections were performed as previously described [21] with slight modifications to normalise infection conditions between the different strains. Briefly, colonies were seeded from fresh agar plates into 3 mls of LB broth with relevant antibiotics and grown with agitation at 37°C overnight. Cultures were then diluted 1∶1000 in DMEM containing 0.3% foetal calf serum and 1 ml added to each well of a 12-well plate. Plates were incubated at 37°C, 5% C0_2_ for 5 hours.

*S.* Typhimurium infections were performed as described [48], with slight modifications to extend the infection time to the same duration as the EPEC infections. Briefly, SL1344 colonies from fresh agar plates were grown in LB broth plus 100 µg/ml ampicillin with agitation at 37°C overnight. Cultures were diluted 1∶33 and grown for a further 4 hours. Infections were performed using 1∶1000 dilutions of these sub-cultures, yielding a multiplicity of infection of 1∶10. Infections were allowed to proceed for 30–40 minutes at 37°C, 5% C0_2_, then washed twice in DMEM+100 µg/ml Gentamycin to remove external bacteria and incubated for a further 4.5 hours at 37°C, 5% C0_2_.

### Immunofluorescence

Following infection, transfected cells were washed in PBS and fixed in 4% formaldehyde solution in PBS for 15 min. Cells were then permeabilised in 0.1% Triton-X 100 in PBS for 2 min, blocked with 10% donkey serum for 15 min and stained using appropriate antibodies for 1 h. Primary antibodies used were anti-Flag (Sigma Aldrich), anti-HA (Covance, USA) and anti-myc 9E10 (Covance, USA). The secondary antibody was Alexa488 or Alexa568-conjugated donkey anti-mouse (Jackson Immunoresearch). Actin was stained with Alexa568 or Alexa488-conjugated phalloidin (Invitrogen), DNA was labelled with DAPI (Invitrogen). Following staining coverslips were washed three times in PBS and mounted in ProLong Gold antifade reagent (Invitrogen). Cells were imaged using a Leica SP5 confocal microscope or a Ziess Axioplan widefield microscope.

### Overexpression screen

Expression constructs in pCMV-SPORT6 were obtained from Open Biosystems. COS-7 cells were transfected with 250 ng of MAL-GFP and 250 ng of expression construct or an empty vector control. Eighteen hours post transfection the cells were washed twice with PBS and incubated in DMEM 0.3% FCS for a further 24 hours. Cells were washed in PBS and fixed in 4% formaldehyde solution in PBS for 15 min, and co-stained with DAPI (Invitrogen). Cellular localization of MAL-GFP was determined by epifluorescence microscopy. Data are the means ± standard deviation of 3 experiments. A minimum of 150 transfected cells were counted for each condition of each experiment.

### Luciferase reporter assays

COS-7 cells were transfected with 50 ng of SRE-luciferase reporter plasmid [8], 1 ng of renilla luciferase (Promega) and either 500 ng of Flag-ABRA/untagged pCMV-ABRA or 150 ng FLRT3-Flag/pCMV-FLRT3. Controls were transfected with the appropriate empty vector. 8 hours post transfection cells were washed twice in PBS and resuspended in DMEM containing 0.3% FCS. 18 hours later cells were lysed in passive lysis buffer (Promega) and luciferase activities were measured with a Glomax 20/20 luminometer (Promega).

### Quantification of F- and G-actin

G-∶F-actin ratios were quantified using a G-actin/F-actin *In vivo* assay kit (Cytoskeleton), in accordance with the manufacturers guidelines. Cells were infected as described above. 3.5 hours post infection samples were washed once in PBS, scraped and lysed with a bent 21 gauge needle in LAS2 lysis buffer. F-actin was then separated from G-actin by centrifugation at 100,000×g for 60 min at 37°C. The F-actin-containing pellet was resuspended in LAS2 buffer containing 2 µM cytochalasin D at a volume equivalent to the G-actin-containing supernatant volume. The resuspended F-actin pellet was kept on ice for 60 min with mixing by pipette every 15 min to dissociate F-actin. The F-actin and G-actin preparations were then assayed for protein. Equal amounts of protein were separated by 10% SDS-PAGE and detected by blotting with anti-actin. Band intensities were quantified with Odyssey application software (LI-COR).

### Accession numbers

The following are the Entrez IDs (http://www.ncbi.nlm.nih.gov/) for the genes discussed in this article.

- *VCL*, 7414
- *SRF*, 6722
- *Cyr61*, 3491
- *Acta2*, 59
- *EGR1*, 1958
- *EGR2*, 1959
- *Fos*, 2353
- *GAPDH*, 2597
- *CFL1*, 1072
- *VAV3*, 10451
- *RSU1*, 6251
- *ARHGDIB*, 397
- *FYN*, 2534
- *FLRT3*, 23767
- *TESK1*, 7016
- *MAL*, 57591
- *VASP*, 7408
- *AMIGO1*, 57463
- *AMIGO2*, 347902
- *TESK2*, 10420
- *FLRT1*, 23769
- *C22orf28*, 51493
- *WDFY3*, 23001
- *SSX2IP*, 117178
- *HIP1R*, 9026
- *WHAMM*, 123720
- *PTP4A3*, 11156
- *ZYXIN*, 7791
- *LPP*, 4026

## Supporting Information

Figure S1 Transcription of a number of SRF Target genes is activated by EPEC infection. Transcription of SRF target genes measured by quantitative polymerase chain reaction (qRT-PCR). Data are the means of at least 3 experiments ± standard deviation.
(0.10 MB TIF)

Figure S2 Exogenous expression of Tir can rescue the EPEC Δ*tir* phenotype. A. MAL-GFP localization in COS-7 cells transfected with TirMC or TirMC Y474F and infected with EPEC Δ*tir* for 5 hours. Data represents the mean of three experiments, where a minimum of 150 transfected cells was counted for each condition of each experiment, ± standard deviation. B. Anti-HA western blot confirming expression of TirMC and TirMC Y474F proteins in COS-7 cells, multiple bands are present due to host modifications of Tir.C. Vector only controls for figure 6E.
(0.53 MB TIF)

Figure S3 cDNA Overexpression-induced nuclear accumulation of MAL-GFP. Immunofluorescence images of MAL-GFP localization in response to overexpression of the indicated cDNAs. COS-7 cells were cotransfected with MAL-GFP and cDNA expression constructs as indicated. After 18 hours they were serum starved for 24 hours then fixed and stained. Scale bar = 20 µm.
(4.51 MB TIF)

Figure S4 Knockdown of ABRA has no significant effect on FLRT3-induced MAL-GFP nuclear accumulation. Data are the means of three experiments ± standard deviation.
(0.23 MB TIF)

Figure S5 SRF localization in EPEC infected COS-7 cells. Scale bar = 20 µm.
(4.25 MB TIF)

Figure S6 Pedestals in ABRA knockdown cells are disorganised. Pedestal formation under microcolonies often leads to large ring structures (arrows) in ABRA knockdown cells.
(4.23 MB TIF)

## References

[1] Hayward RD, Leong JM, Koronakis V, Campellone KG (2006). Exploiting pathogenic Escherichia coli to model transmembrane receptor signalling.. Nat Rev Microbiol.

[2] Stebbins CE, Galan JE (2001). Structural mimicry in bacterial virulence.. Nature.

[3] Bhavsar AP, Guttman JA, Finlay BB (2007). Manipulation of host-cell pathways by bacterial pathogens.. Nature.

[4] Cornelis GR (2010). The type III secretion injectisome, a complex nanomachine for intracellular ‘toxin’ delivery.. Biol Chem.

[5] Treisman R (1990). The SRE: a growth factor responsive transcriptional regulator.. Semin Cancer Biol.

[6] Chai J, Tarnawski AS (2002). Serum response factor: discovery, biochemistry, biological roles and implications for tissue injury healing.. J Physiol Pharmacol.

[7] Posern G, Treisman R (2006). Actin' together: serum response factor, its cofactors and the link to signal transduction.. Trends Cell Biol.

[8] Hill CS, Wynne J, Treisman R (1995). The Rho family GTPases RhoA, Rac1, and CDC42Hs regulate transcriptional activation by SRF.. Cell.

[9] Miralles F, Posern G, Zaromytidou AI, Treisman R (2003). Actin dynamics control SRF activity by regulation of its coactivator MAL.. Cell.

[10] Vartiainen MK, Guettler S, Larijani B, Treisman R (2007). Nuclear actin regulates dynamic subcellular localization and activity of the SRF cofactor MAL.. Science.

[11] Grosse R, Copeland JW, Newsome TP, Way M, Treisman R (2003). A role for VASP in RhoA-Diaphanous signalling to actin dynamics and SRF activity.. EMBO J.

[12] Boquet P, Lemichez E (2003). Bacterial virulence factors targeting Rho GTPases: parasitism or symbiosis?. Trends Cell Biol.

[13] Jaffe AB, Hall A (2005). Rho GTPases: biochemistry and biology.. Annu Rev Cell Dev Biol.

[14] Kuwahara K, Barrientos T, Pipes GC, Li S, Olson EN (2005). Muscle-specific signaling mechanism that links actin dynamics to serum response factor.. Mol Cell Biol.

[15] Tabuchi A, Estevez M, Henderson JA, Marx R, Shiota J (2005). Nuclear translocation of the SRF co-activator MAL in cortical neurons: role of RhoA signalling.. J Neurochem.

[16] Du KL, Chen M, Li J, Lepore JJ, Mericko P (2004). Megakaryoblastic leukemia factor-1 transduces cytoskeletal signals and induces smooth muscle cell differentiation from undifferentiated embryonic stem cells.. J Biol Chem.

[17] Campellone KG, Giese A, Tipper DJ, Leong JM (2002). A tyrosine-phosphorylated 12-amino-acid sequence of enteropathogenic Escherichia coli Tir binds the host adaptor protein Nck and is required for Nck localization to actin pedestals.. Mol Microbiol.

[18] Campellone KG, Rankin S, Pawson T, Kirschner MW, Tipper DJ (2004). Clustering of Nck by a 12-residue Tir phosphopeptide is sufficient to trigger localized actin assembly.. J Cell Biol.

[19] Campellone KG, Leong JM (2005). Nck-independent actin assembly is mediated by two phosphorylated tyrosines within enteropathogenic Escherichia coli Tir.. Mol Microbiol.

[20] Garmendia J, Phillips AD, Carlier MF, Chong Y, Schuller S (2004). TccP is an enterohaemorrhagic Escherichia coli O157:H7 type III effector protein that couples Tir to the actin-cytoskeleton.. Cell Microbiol.

[21] Campellone KG, Robbins D, Leong JM (2004). EspFU is a translocated EHEC effector that interacts with Tir and N-WASP and promotes Nck-independent actin assembly.. Dev Cell.

[22] Vingadassalom D, Kazlauskas A, Skehan B, Cheng HC, Magoun L (2009). Insulin receptor tyrosine kinase substrate links the E. coli O157:H7 actin assembly effectors Tir and EspF(U) during pedestal formation.. Proc Natl Acad Sci U S A.

[23] Weiss SM, Ladwein M, Schmidt D, Ehinger J, Lommel S (2009). IRSp53 links the enterohemorrhagic E. coli effectors Tir and EspFU for actin pedestal formation.. Cell Host Microbe.

[24] Arai A, Spencer JA, Olson EN (2002). STARS, a striated muscle activator of Rho signaling and serum response factor-dependent transcription.. J Biol Chem.

[25] Hu J, Teng J, Ding N, He M, Sun Y (2008). FAAP, a novel murine protein, is involved in cell adhesion through regulating vinculin-paxillin association.. Front Biosci.

[26] Toshima J, Toshima JY, Amano T, Yang N, Narumiya S (2001). Cofilin phosphorylation by protein kinase testicular protein kinase 1 and its role in integrin-mediated actin reorganization and focal adhesion formation.. Mol Biol Cell.

[27] Tsuji L, Yamashita T, Kubo T, Madura T, Tanaka H (2004). FLRT3, a cell surface molecule containing LRR repeats and a FNIII domain, promotes neurite outgrowth.. Biochem Biophys Res Commun.

[28] Chen X, Koh E, Yoder M, Gumbiner BM (2009). A protocadherin-cadherin-FLRT3 complex controls cell adhesion and morphogenesis.. PLoS One.

[29] Ng AC, Eisenberg JM, Heath RJ, Huett A, Robinson CM (2010). Microbes and Health Sackler Colloquium: Human leucine-rich repeat proteins: a genome-wide bioinformatic categorization and functional analysis in innate immunity.. Proc Natl Acad Sci U S A.

[30] Patel JC, Galan JE (2006). Differential activation and function of Rho GTPases during Salmonella-host cell interactions.. J Cell Biol.

[31] McGhie EJ, Brawn LC, Hume PJ, Humphreys D, Koronakis V (2009). Salmonella takes control: effector-driven manipulation of the host.. Curr Opin Microbiol.

[32] Fu Y, Galan JE (1999). A salmonella protein antagonizes Rac-1 and Cdc42 to mediate host-cell recovery after bacterial invasion.. Nature.

[33] Rangnekar VM, Aplin AC, Sukhatme VP (1990). The serum and TPA responsive promoter and intron-exon structure of EGR2, a human early growth response gene encoding a zinc finger protein.. Nucleic Acids Res.

[34] Fujii M, Niki T, Mori T, Matsuda T, Matsui M (1991). HTLV-1 Tax induces expression of various immediate early serum responsive genes.. Oncogene.

[35] Kobayashi SD, Braughton KR, Whitney AR, Voyich JM, Schwan TG (2003). Bacterial pathogens modulate an apoptosis differentiation program in human neutrophils.. Proc Natl Acad Sci U S A.

[36] Phelps ED, Sweeney KR, Blader IJ (2008). Toxoplasma gondii rhoptry discharge correlates with activation of the early growth response 2 host cell transcription factor.. Infect Immun.

[37] Lee SM, Vasishtha M, Prywes R (2010). Activation and repression of cellular immediate early genes by serum response factor cofactors.. J Biol Chem.

[38] Boldrick JC, Alizadeh AA, Diehn M, Dudoit S, Liu CL (2002). Stereotyped and specific gene expression programs in human innate immune responses to bacteria.. Proc Natl Acad Sci U S A.

[39] Kenny B, DeVinney R, Stein M, Reinscheid DJ, Frey EA (1997). Enteropathogenic E. coli (EPEC) transfers its receptor for intimate adherence into mammalian cells.. Cell.

[40] Kenny B (1999). Phosphorylation of tyrosine 474 of the enteropathogenic Escherichia coli (EPEC) Tir receptor molecule is essential for actin nucleating activity and is preceded by additional host modifications.. Mol Microbiol.

[41] Phillips N, Hayward RD, Koronakis V (2004). Phosphorylation of the enteropathogenic E. coli receptor by the Src-family kinase c-Fyn triggers actin pedestal formation.. Nat Cell Biol.

[42] Swimm A, Bommarius B, Li Y, Cheng D, Reeves P (2004). Enteropathogenic Escherichia coli use redundant tyrosine kinases to form actin pedestals.. Mol Biol Cell.

[43] Gruenheid S, DeVinney R, Bladt F, Goosney D, Gelkop S (2001). Enteropathogenic E. coli Tir binds Nck to initiate actin pedestal formation in host cells.. Nat Cell Biol.

[44] DeVinney R, Puente JL, Gauthier A, Goosney D, Finlay BB (2001). Enterohaemorrhagic and enteropathogenic Escherichia coli use a different Tir-based mechanism for pedestal formation.. Mol Microbiol.

[45] Kenny B (2001). The enterohaemorrhagic Escherichia coli (serotype O157:H7) Tir molecule is not functionally interchangeable for its enteropathogenic E. coli (serotype O127:H6) homologue.. Cell Microbiol.

[46] DeVinney R, Stein M, Reinscheid D, Abe A, Ruschkowski S (1999). Enterohemorrhagic Escherichia coli O157:H7 produces Tir, which is translocated to the host cell membrane but is not tyrosine phosphorylated.. Infect Immun.

[47] Herrera RE, Shaw PE, Nordheim A (1989). Occupation of the c-fos element in vivo is unaltered by growth factor induction.. Nature.

[48] Rioux JD, Xavier RJ, Taylor KD, Silverberg MS, Goyette P (2007). Genome-wide association study identifies new susceptibility loci for Crohn disease and implicates autophagy in disease pathogenesis.. Nat Genet.

