# Supplementary figures and images for: Bacterial and Host Determinants of MAL Activation upon EPEC Infection: The Roles of Tir, ABRA, and FLRT3
Source: PLoS Pathog. 2011 Apr 7;7(4):e1001332. doi: 10.1371/journal.ppat.1001332 (PMC3072376; doi:10.1371/journal.ppat.1001332)

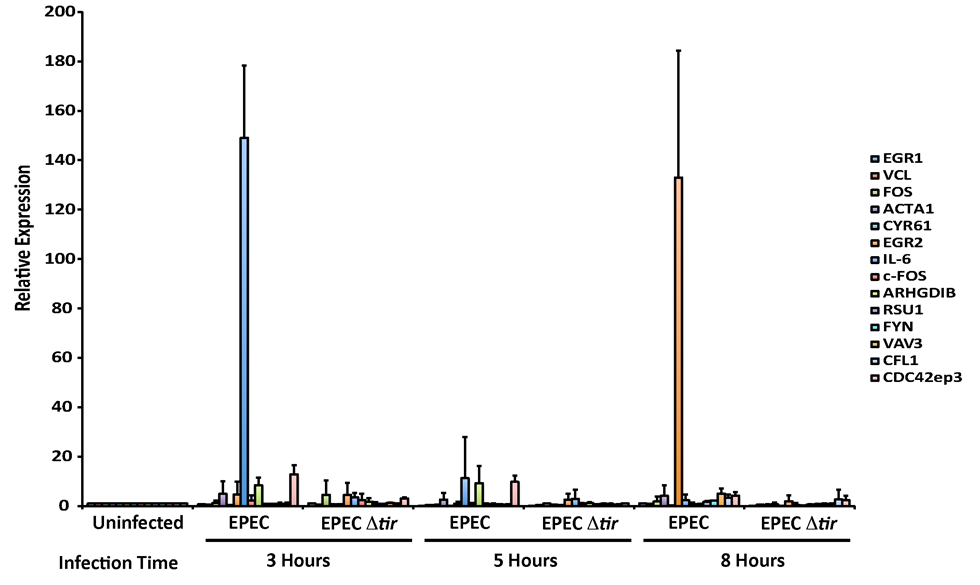

Supplement: Figure S1 — Transcription of a number of SRF Target genes is activated by EPEC infection. Transcription of SRF target genes measured by quantitative polymerase chain reaction (qRT-PCR). Data are the means of at least 3 experiments ± standard deviation. (0.10 MB TIF) [file ppat.1001332.s001.tif]

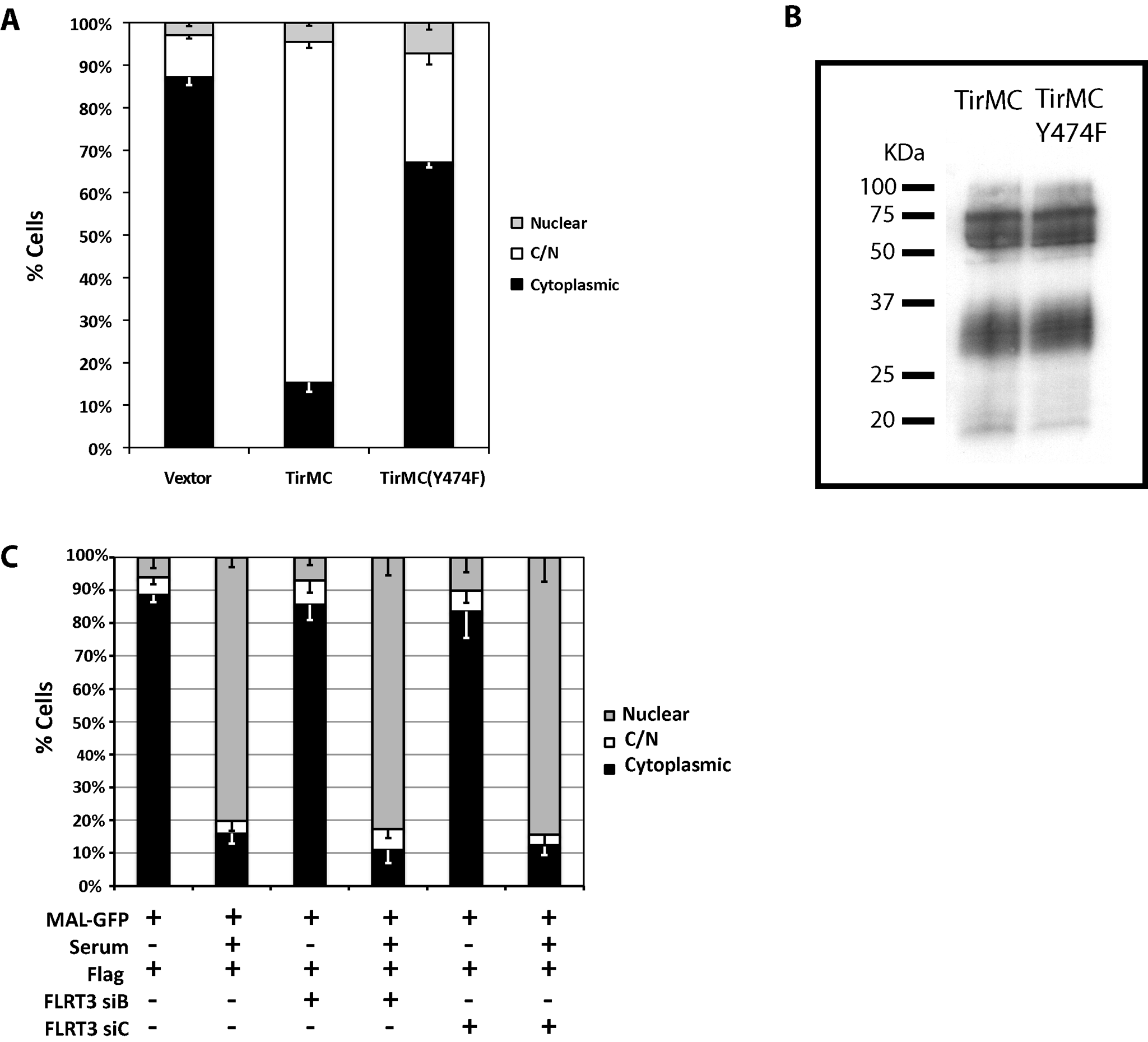

Supplement: Figure S2 — Exogenous expression of Tir can rescue the EPEC Δtir phenotype. A. MAL-GFP localization in COS-7 cells transfected with TirMC or TirMC Y474F and infected with EPEC Δtir for 5 hours. Data represents the mean of three experiments, where a minimum of 150 transfected cells was counted for each condition of each experiment, ± standard deviation. B. Anti-HA western blot confirming expression of TirMC and TirMC Y474F proteins in COS-7 cells, multiple bands are present due to host modifications of Tir.C. Vector only controls for figure 6E. (0.53 MB TIF) [file ppat.1001332.s002.tif]

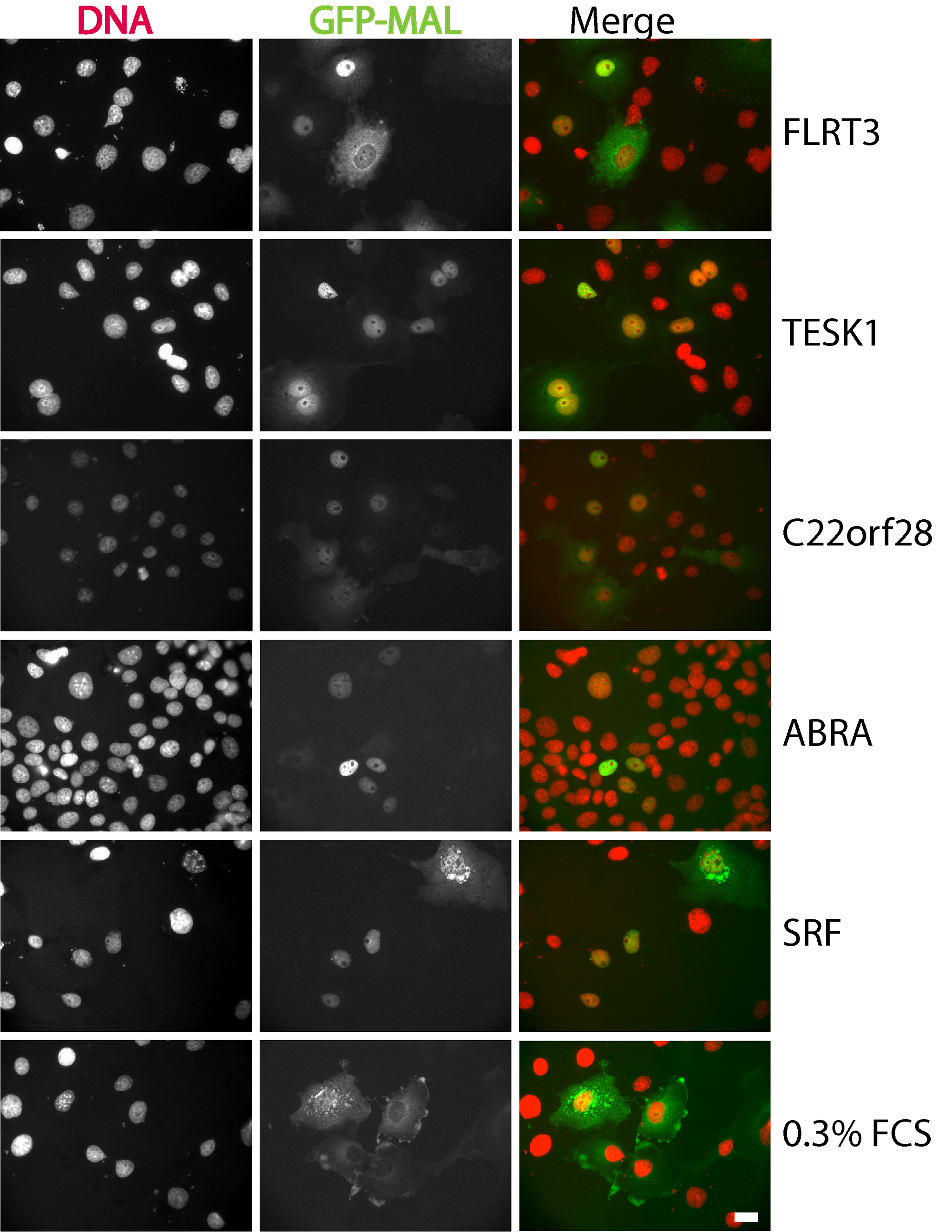

Supplement: Figure S3 — cDNA Overexpression-induced nuclear accumulation of MAL-GFP. Immunofluorescence images of MAL-GFP localization in response to overexpression of the indicated cDNAs. COS-7 cells were cotransfected with MAL-GFP and cDNA expression constructs as indicated. After 18 hours they were serum starved for 24 hours then fixed and stained. Scale bar = 20 µm. (4.51 MB TIF) [file ppat.1001332.s003.tif]

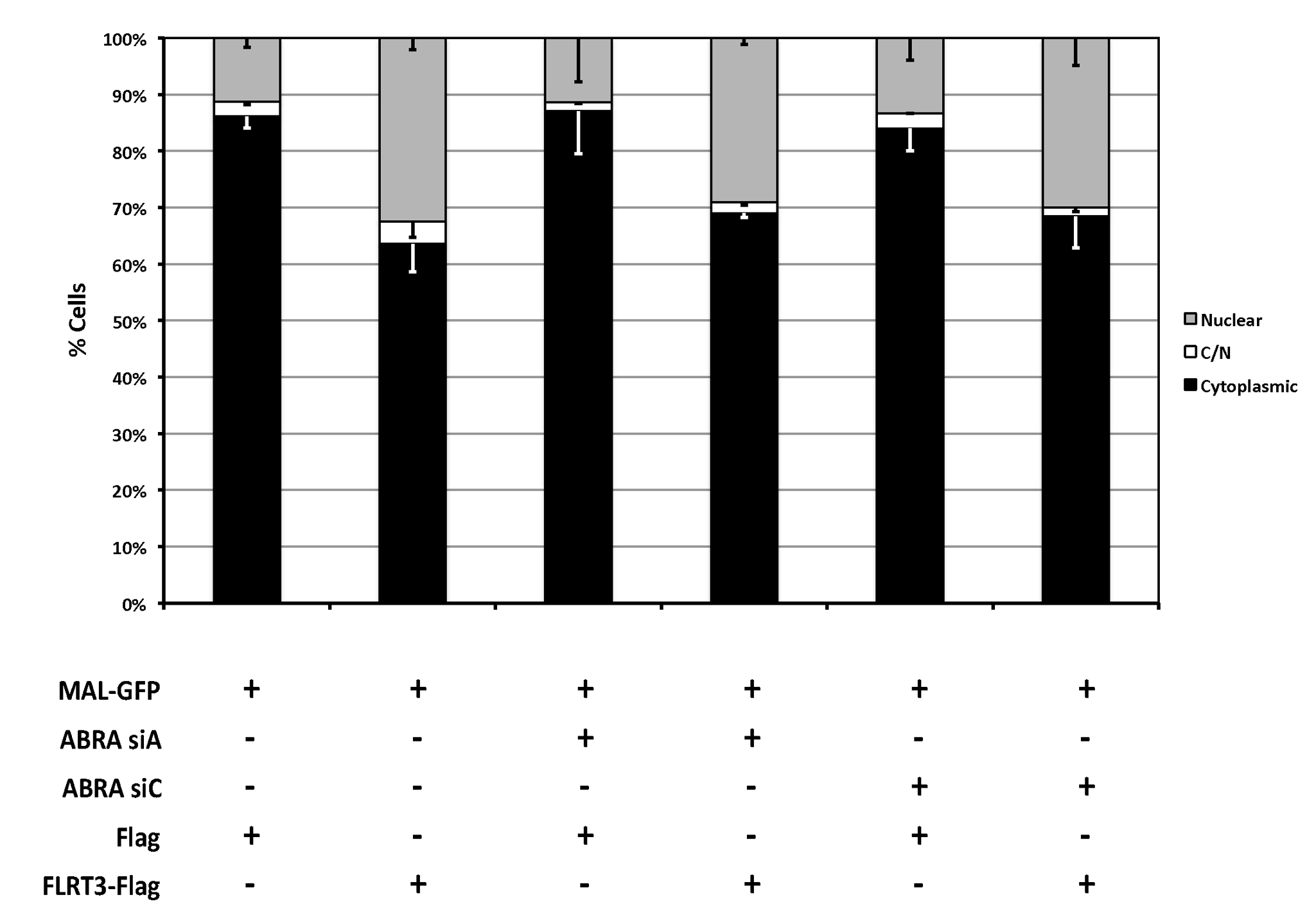

Supplement: Figure S4 — Knockdown of ABRA has no significant effect on FLRT3-induced MAL-GFP nuclear accumulation. Data are the means of three experiments ± standard deviation. (0.23 MB TIF) [file ppat.1001332.s004.tif]

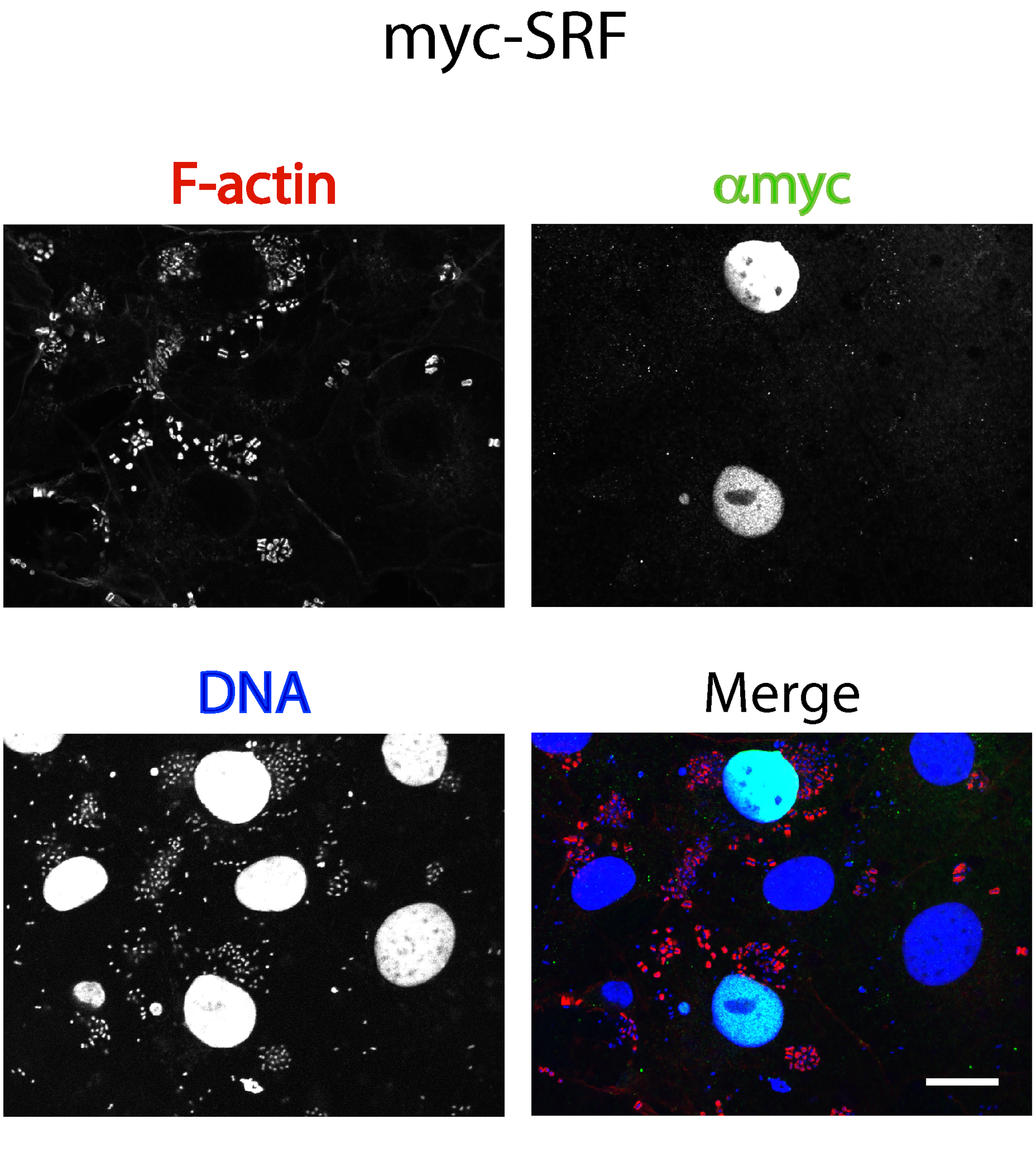

Supplement: Figure S5 — SRF localization in EPEC infected COS-7 cells. Scale bar = 20 µm. (4.25 MB TIF) [file ppat.1001332.s005.tif]

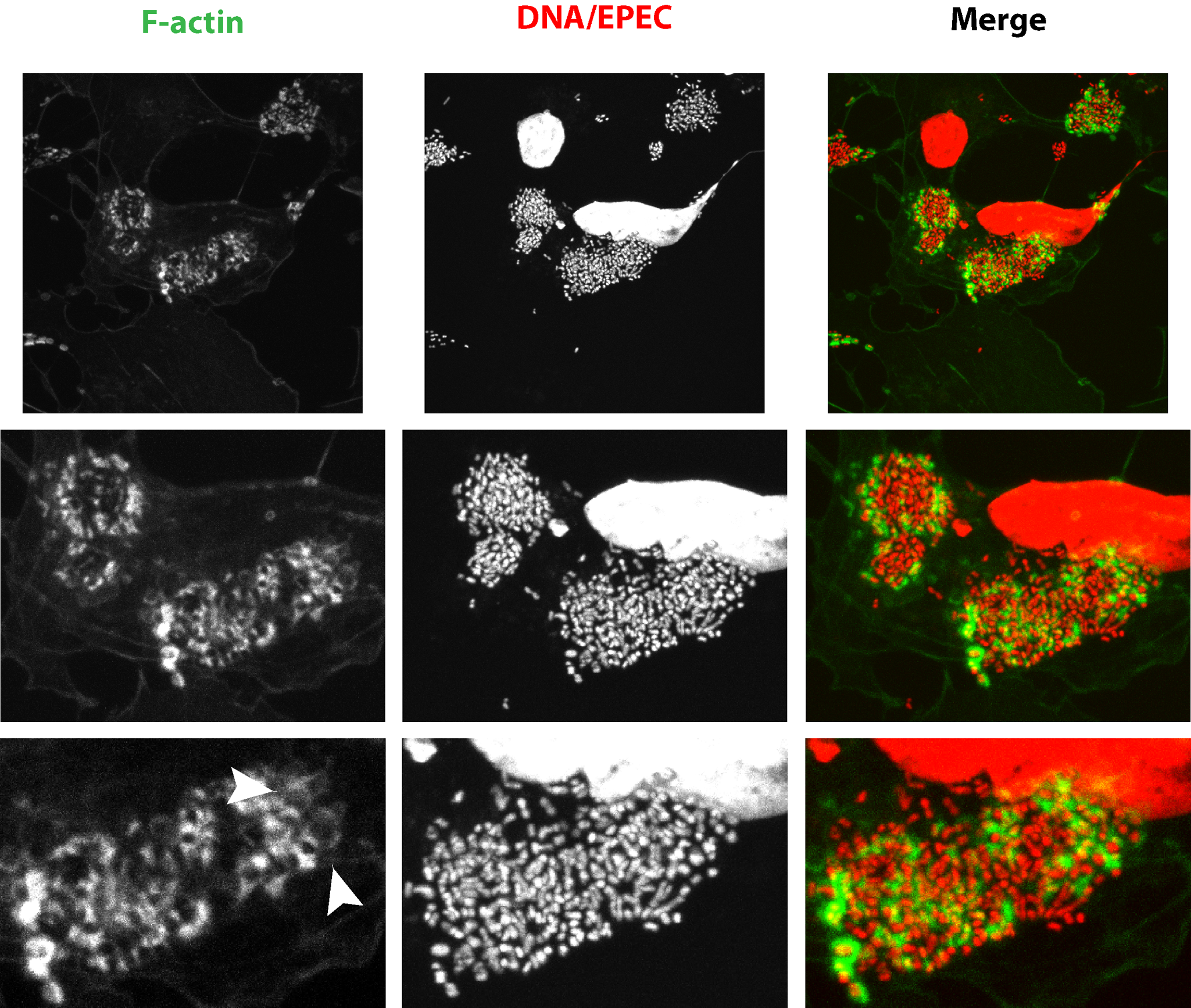

Supplement: Figure S6 — Pedestals in ABRA knockdown cells are disorganised. Pedestal formation under microcolonies often leads to large ring structures (arrows) in ABRA knockdown cells. (4.23 MB TIF) [file ppat.1001332.s006.tif]
